# Heterogeneous Histories of Recombination Suppression on Stickleback Sex Chromosomes

**DOI:** 10.1093/molbev/msab179

**Published:** 2021-06-12

**Authors:** Jason M Sardell, Matthew P Josephson, Anne C Dalziel, Catherine L Peichel, Mark Kirkpatrick

**Affiliations:** 1 Department of Integrative Biology, University of Texas at Austin, Austin, TX, USA; 2 Institute of Ecology and Evolution, University of Bern, Bern, Switzerland; 3 Department of Biology, Saint Mary’s University, Halifax, NS, Canada

**Keywords:** sex chromosomes, stickleback, *Gasterosteus*, neo-Y chromosome, recombination, fish

## Abstract

How consistent are the evolutionary trajectories of sex chromosomes shortly after they form? Insights into the evolution of recombination, differentiation, and degeneration can be provided by comparing closely related species with homologous sex chromosomes. The sex chromosomes of the threespine stickleback (*Gasterosteus aculeatus*) and its sister species, the Japan Sea stickleback (*G. nipponicus*), have been well characterized. Little is known, however, about the sex chromosomes of their congener, the blackspotted stickleback (*G. wheatlandi*). We used pedigrees to obtain experimentally phased whole genome sequences from blackspotted stickleback X and Y chromosomes. Using multispecies gene trees and analysis of shared duplications, we demonstrate that Chromosome 19 is the ancestral sex chromosome and that its oldest stratum evolved in the common ancestor of the genus. After the blackspotted lineage diverged, its sex chromosomes experienced independent and more extensive recombination suppression, greater X–Y differentiation, and a much higher rate of Y degeneration than the other two species. These patterns may result from a smaller effective population size in the blackspotted stickleback. A recent fusion between the ancestral blackspotted stickleback Y chromosome and Chromosome 12, which produced a neo-X and neo-Y, may have been favored by the very small size of the recombining region on the ancestral sex chromosome. We identify six strata on the ancestral and neo-sex chromosomes where recombination between the X and Y ceased at different times. These results confirm that sex chromosomes can evolve large differences within and between species over short evolutionary timescales.

## Introduction

Sex chromosomes across the tree of life vary greatly in their levels of differentiation and degeneration (Bachtrog et al. 2014; Beukeboom and Perrin 2014). The Y chromosomes of mammals and the W chromosomes of birds have degenerated greatly since they ceased to recombine over 140 Ma (Cortez et al. 2014). At the other extreme, the X and Y chromosomes of the fugu (*Takifugu rubripes*), which are 1 My old, differ by only a single nucleotide (Kamiya et al. 2012). In between these extremes lie a host of other patterns. For example, some *Rana* frogs have degenerate Y chromosomes, whereas others have Ys that are nearly indistinguishable from the X, and variation can occur within populations of the same species (Jeffries et al. 2018; Toups et al. 2019; Phillips et al. 2020). Many *Drosophila* possess neo-sex chromosomes, but only some of those neo-Ys have degenerated (Vicoso and Bachtrog 2015). Finally, most dioecious plants have homomorphic sex chromosomes with small SDRs (Renner and Muller 2021), but the Y chromosome of some (e.g., *Silene latifolia* and *Rumex hastatulus*) have degenerated extensively (Papadopulos et al. 2015).

Genomic comparisons of sex chromosomes of closely related species could provide clues to the origins of these kinds of dramatic differences. For example, do differences in the extent of degeneration arise between closely related species because they independently evolved sex chromosomes at different times in the past? Or do they harbor homologous sex chromosomes that evolved with different rates of recombination suppression and gene loss?

Stickleback fishes (family Gasterosteidae) present an opportunity to address these questions, as they show variation in sex determination among closely related species. They have experienced sex chromosome turnovers (Ross et al. 2009), transitions between XY and ZW sex determination (Chen and Reisman 1970; Ross et al. 2009; Natri et al. 2019), fusions between sex chromosomes and autosomes (Kitano et al. 2009; Ross et al. 2009; Yoshida et al. 2014; Dagilis 2019), and the origin of a sex chromosome by introgression (Natri et al. 2013; Dixon et al. 2019). As a result, nearly every species possesses a different sex chromosome system.

**Fig. 1.**
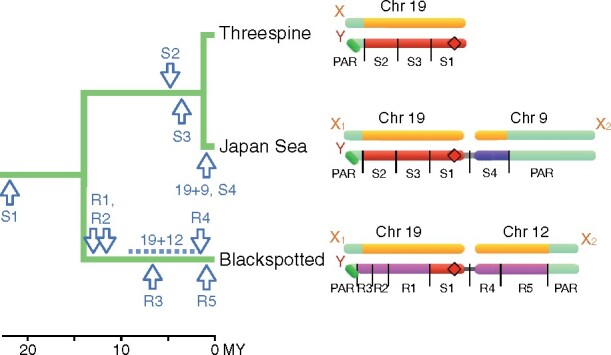
Left: The phylogeny and major events in the evolution of the sex chromosomes in the three species of *Gasterosteus* sticklebacks. Estimated dates are shown for the fusions (19 + 9 and 19 + 12) as well as the origins of the strata. Right: Schematics of the sex chromosomes in the sticklebacks showing their strata (indicated with S or R) and PARs, with sizes to scale. The coordinates are based on mapping to the X chromosome of the threespine reference genome, which lacks a Y chromosome. Strata that are homologous across species are shown in the same color. Only stratum S1 is homologous in all three species. The location of the sex-determining gene in the threespine stickleback is shown by diamonds. The data for the blackspotted stickleback are presented in this article.

The focus of this study is the blackspotted stickleback, *Gasterosteus wheatlandi*. Figure 1 shows the physical organization of the sex chromosomes in this and the two other species in the genus, and the major events in their evolution based on previous work and this study. Despite frequent turnovers in other stickleback genera, Chr 19 determines sex in all three extant species of *Gasterosteus* sticklebacks (Peichel et al. 2004; Kitano et al. 2009; Ross et al. 2009). The sex chromosomes in the threespine stickleback (*G. aculeatus*) have been well characterized (Ross and Peichel 2008; Leder et al. 2010; Roesti et al. 2013; Schultheiß et al. 2015; White et al. 2015; Peichel et al. 2020). They include a 2.5 Mb pseudoautosomal region (PAR) in which the X and Y continue to recombine (fig. 1). Recombination between the X and Y is suppressed over a 16 Mb sex-determining region (SDR). It contains three “strata,” which are regions where recombination was suppressed at different times in the past and correspond to known inversions (Ross and Peichel 2008; Roesti et al. 2013; White et al. 2015; Peichel et al. 2020). The oldest stratum (S1), which Peichel et al. (2020) estimated stopped recombining 22 Ma, is highly degenerate. The younger two strata (S2 and S3), which stopped recombining between 4.7 and 6 Ma, are less degenerate. The Japan Sea stickleback (*G. nipponicus*), which is sister to the threespine stickleback, shares those three strata on Chr 19, indicating that recombination ceased in their common ancestor (Dagilis 2019). No substantial differences between the two species have evolved on Chr 19 in the 2 My since they diverged. However, the Y on Chr 19 in the Japan Sea stickleback fused with and autosome, Chr 9. The Chr 9 neo-Y chromosome now carries an SDR of 6.9 Mb that has experienced little degeneration since it stopped recombining less than 2 Ma (Kitano et al. 2009; Natri et al. 2013; Yoshida et al. 2014, 2017; Dagilis 2019).

Much less is known about sex chromosomes in the blackspotted stickleback (*G. wheatlandi*), which split from the ancestor of threespine and Japan Sea stickleback about 14 Ma (Varadharajan et al. 2019). Using cytogenetics, Ross et al. (2009) showed that the blackspotted Y chromosome comprises a fusion between Chr 19 and Chr 12. They failed to amplify Y-linked alleles for any of five microsatellites on the threespine Chr 19, suggesting that a large region of the SDR on this chromosome has degenerated. Four Y-linked microsatellites on Chr 12 did amplify, suggesting that this chromosome became sex-linked more recently and has not degenerated to the same extent as the SDR on Ch 19. The blackspotted stickleback has not, however, been studied at the genomic level. Thus, much remains unknown about the evolutionary history of its sex chromosomes. For example, how much variation is there along the blackspotted stickleback Y chromosome in the extent of degeneration and differentiation with the X, and how does its Y compare with those of the threespine and Japan Sea sticklebacks? Did evolutionary strata independently evolve repressed recombination in the blackspotted stickleback, or did they evolve in the ancestor of all extant *Gasterosteus*? Is Chr 19 was the ancestral sex chromosome of all three species? This last question is pertinent because Chr 12 determines sex in the more distantly related ninespine stickleback (*Pungitius pungitius*), so that chromosome is another candidate for the ancestral sex chromosome of the genus *Gasterosteus* (Ross et al. 2009; Shapiro et al. 2009; Dixon et al. 2019; Natri et al. 2019).

**Fig. 2.**
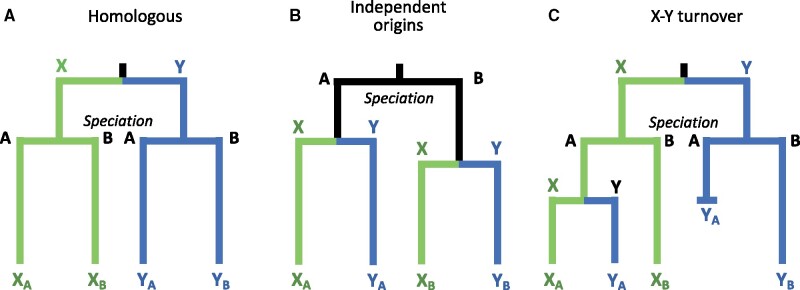
Different evolutionary histories of the SDRs on Y chromosomes result in different multispecies gene tree topologies. (*A*) Topology when the SDRs on the Ys of two species (A and B) are homologous, having originated in their common ancestor. (*B*) Topology when the SDRs on the Y chromosomes originated independently in two species. (*C*) Topology when a turnover event in Species A generates a new SDR on the Y from the X chromosome or SDR, whereas the SDR in Species B remains unchanged.

To compare rates of evolution across these species, it is critical to determine if their Y chromosomes are homologous, by which we mean that they descended from the Y of their common ancestor and did not originate independently. (Note that other authors refer to sex chromosomes as homologous in different species if they are in the same linkage group, regardless of their evolutionary histories.) Homology controls for the effects of evolutionary time, as sex chromosome turnovers “reset the clock” of Y chromosome evolution. Observing that the same chromosome pair determines sex is not sufficient to establish homology, however, as the same autosome can be independently recruited as a sex chromosome in closely related species (Montiel et al. 2017; Jeffries et al. 2018; Rovatsos et al. 2019). In other cases, a copy of the X chromosome has independently evolved into a new Y chromosome, making the Y chromosomes of closely related species nonhomologous (Blaser et al. 2013; Meisel et al. 2017; Ieda et al. 2018) (fig. 2). Y chromosome homology can be inferred from multispecies gene tree topologies in which the Y chromosomes of all species form a single clade to the exclusion of the X chromosomes (García-Moreno and Mindell 2000; Handley et al. 2004; Natri et al. 2013; Dixon et al. 2019; Darolti et al. 2020). Shared chromosomal rearrangements such as duplications, insertions, or inversions on the sex chromosomes also provide further evidence of homology. Finally, homologous Y chromosomes can have nonhomologous strata if suppressed recombination evolved independently in the two species.

Here, we present the first genomic investigation of the blackspotted stickleback sex chromosomes. Using whole genome sequencing of parents and offspring in 15 families, we obtained experimentally phased sequences from 15 X and 15 Y chromosomes. We find that the blackspotted stickleback SDR extends along nearly the entire length of Chr 19, and that the Y is extremely degenerated over this whole region. This situation contrasts with that in threespine and Japan Sea sticklebacks, where extreme degeneration is limited to the oldest stratum on Chr 19 (S1 in fig. 1). We demonstrate that this stratum is homologous and is the ancestral SDR in the three *Gasterosteus* sticklebacks, and that it has degenerated much more rapidly in the blackspotted stickleback. Although the younger strata evolved independently in the blackspotted stickleback and the ancestor of threespine and Japan Sea sticklebacks, suppressed recombination evolved much more rapidly in blackspotted stickleback. The fused neo-Y of Chr 12 in blackspotted stickleback has an SDR with little degeneration, but it is much larger than the neo-Y on Chr 9 in Japan Sea stickleback, which is of similar age. These results demonstrate that homologous sex chromosomes can evolve striking differences over short evolutionary timescales.

## Results

We obtained phased sequences of X and Y chromosomes using 15 small pedigrees. These comprised interspecific crosses, each consisting of a blackspotted stickleback father, a threespine stickleback mother, one daughter, and one son (supplementary fig. S1, Supplementary Material online). Using interspecific crosses increases genetic divergence between the two sex chromosomes of the offspring, which improves the power to phase. All four members of each family were whole genome sequenced, and the haploid genome sequences of the blackspotted father’s X-bearing and Y-bearing sperm were determined from patterns of transmission (see also, Sardell et al. 2018; Dagilis 2019). In this way, we obtained the sequences of 15 independent X chromosomes and 15 independent Y chromosomes from the blackspotted stickleback. All reads were mapped to the repeat-masked version of the threespine stickleback reference genome (Glazer et al. 2015). This reference was produced from a female and so lacks a sequence for the Y. We did not map reads to the recently published threespine stickleback Y reference sequences (Peichel et al. 2020). We suspected (and later confirmed) that the blackspotted and threespine independently evolved suppressed recombination, so using the threespine Y would confound our analyses (see Sequence Assembly and SNP Calling in the Materials and Methods section). All genome positions reported here from the blackspotted stickleback refer to coordinates of the threespine reference.

**Fig. 3.**
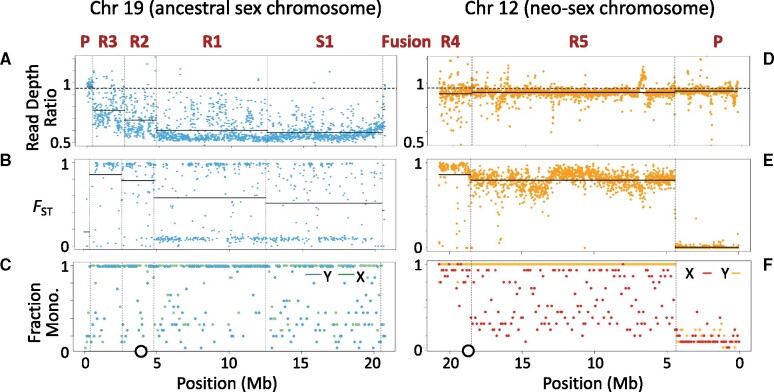
Population genetic statistics for the sex chromosomes in the blackspotted stickleback sex chromosomes. Dashed vertical lines show boundaries between the PAR (labeled P) and the strata in the SDR (labeled S1 and R1 to R5, in order of decreasing age) on the ancestral sex chromosome (Chr 19; left) and neo-sex chromosome (Chr 12; right). (*A* and *D*) Plot of mean male/female read depth ratio. Dashed horizontal gray line represents the autosomal mean, and solid horizontal lines are the means for each stratum. (*B* and *E*) *F*_ST_ between 15 phased X and 15 phased Y chromosomes. Solid horizontal lines are the means for each stratum. (*C* and *F*) Fraction of Y (or X) chromosomes that fall within the largest monophyletic clade of Y (or X) chromosomes on the gene tree. Dots are averages in 10 kb windows. The circles on the X axis of the lower panels show the locations of the centromeres on Chr 19 and Chr 12 in threespine stickleback.

Three patterns of molecular variation are often seen in SDRs, all of which are found in blackspotted stickleback (fig. 3). First, the X and Y typically show elevated genetic differentiation (measured, e.g., by *F*_ST_) because lack of recombination allows the two sex chromosomes to accumulate different substitutions (Vicoso and Bachtrog 2015; Palmer et al. 2019). Second, the ratio of read depths in males versus females (the read depth ratio) often drops below one (Vicoso and Bachtrog 2015; Palmer et al. 2019). This results as the Y degenerates with the accumulation of deletions (making the X hemizygous in males) and when the remaining regions of the Y diverge so much that reads from it do not map to a genome reference sequence that has only the X.

The third pattern seen in SDRs is Y monophyly (Natri et al. 2013; Dixon et al. 2019; Toups et al. 2019). When an SDR establishes or expands via a *cis* modifier (e.g., an inversion on the Y chromosome), the SDRs on all Ys in the population descend from a single ancestral Y. Consequently, all Y sequences are monophyletic with respect to the X sequences in gene trees from this region. If enough time has passed since recombination ceased, the X chromosomes will also be monophyletic. The argument works conversely when recombination is suppressed via an X-linked modifier. By contrast, recombination in the PAR causes the Xs and Ys to be intermingled on the gene tree for that region of the sex chromosome. The monophyly criterion is a sensitive test for recombination because departure from monophyly is the cumulative result of many generations of recombination.

### The SDR on the Ancestral Sex Chromosome (Chr 19)

In a later section (Multispecies Gene Trees), we will establish that a stratum on Chr 19 is the ancestral SDR for all three species, and for convenience we will refer to it here as such. Both the read depth ratio and the monophyly criterion show that the SDR spans nearly all of the ancestral sex chromosome (Chr 19) in blackspotted stickleback (fig. 3). Most SNPs have a male/female read depth ratio nearer to 0.5 than to 1.0 (supplementary fig. S3, Supplementary Material online), indicating extensive degeneration on the Y. *F*_ST_ between the X and Y is also highly elevated over nearly the entire chromosome (figs. 3*B*), suggesting a lack of recombination.

A small region (400 kb, or about 2% of the chromosome) at the end of Chr 19 distal to the fusion is a PAR (figs. 1 and 3). The gene trees in this region do not exhibit X or Y monophyly, showing that it continues to recombine (fig. 3*C*). The read depth ratio is near 1 here (fig. 3*A*), and most windows have low *F*_ST_ between the X and Y (fig. 3*B*).

Among the 100 kb windows in the SDR, 74% (150/203) exhibit X monophyly, as expected if recombination between the X and Y ceased long ago (fig. 3*C*). Patterns are noisier on the Y, with only 46% (94/203) of windows in its SDR exhibiting monophyly, likely because of genotyping and phasing errors. These errors are more frequent in regions with high Y degeneration because hemizygosity in males causes SNP calling and phasing algorithms to erroneously impute X alleles onto the Y. We applied several bioinformatic filters to discard hemizygous regions when testing for monophyly, but some may still occur in our data (see Materials and Methods). Windows with incomplete Y monophyly might also result from gene conversion between the X and Y, if it exists, and in any event that is expected to be so rare as to not affect the basic pattern observed.

Along the length of the SDR, we see that read depth ratios vary, suggesting the presence of strata (fig. 3*A*). We identified the boundaries of four putative strata on Chr 19 using two methods: an algorithm that detects changepoints in the read depth ratio (Killick and Eckley 2014) and multispecies gene trees (see Multispecies Gene Trees below). The boundaries and several statistics for the strata are summarized in table 1. We emphasize that the positions and ordering of all the blackspotted strata shown in the figures are based on the X chromosome coordinates of the threespine reference. Their actual locations on the blackspotted Y are likely to have been altered by inversions and deletions.

**Table 1.** Statistics for Strata in Blackspotted Stickleback on the Ancestral Sex Chromosome (Chr 19) and the Neo-Sex Chromosome (Chr 12).

Region	Position (Mb)	No. of Genes on X	% Genes Deleted from Y	Age (Ma)	*d*_S_ Y_BS_ versus X_BS_	*d*_N_ Y_BS_ versus X_BS_	*d*_N_*/d*_S_ Y_BS_ versus X_BS_	*d*_S_ Y_BS_ versus X_TS_
Chr 19								
P	0–0.4	7	0	0	0	0	n/a	0.004
R3	0.4–2.6	109	70	7.2	0.030	0.007	0.24	0.060
R2	2.6–4.8	78	77	12	0.062	0.014	0.25	0.069
R1	4.8–12.5	326	83	13	0.050	0.015	0.36	0.058
S1	12.5–20.6	443	92	22^a^	0.090	0.016	0.20	0.078
Chr 12								
R4	18.5–20.8	183	8	1.7	0.012	0.003	0.33	0.099
R5	4.4–18.5	1,379	1.8	1.0	0.004	0.001	0.38	0.065
P	0–4.4	363	0.8	0	0.0004	0.0002	n/a	0.075

Note.—Positions are based on the reference genome by Glazer et al. (2015). For *d*_S_ between the blackspotted Y and X, the significant comparisons (*P *<* *0.005, Mann–Whitney *U* test) between strata are: S1 versus R1, S1 versus R3, and R1 versus R3 on Chr 19, and R4 versus R5 on Chr 12. For *d*_N_ between the blackspotted Y and X, the significant comparisons (*P *<* *0.005, Mann–Whitney *U* test) between strata are: S1 versus R3 and R1 versus R3 on Chr 19, and R4 versus R5 on Chr 12. No comparison between *d*_N_*/d*_S_ ratios is significant. Lack of significant comparisons involving R2 likely reflects the small number of genes in that stratum. X_BS_, Y_BS_, X and Y chromosomes in blackspotted stickleback; X_TS_, X chromosomes in threespine stickleback for Chr 19 (or autosome for Chr 12).

a Age of S1 taken from Peichel et al. (2020).

Stratum S1 is the oldest, and we show later in this paper that it is homologous to the oldest stratum on the threespine Y (Roesti et al. 2013; White et al. 2015; Peichel et al. 2020). Thus the inversion that established the stratum fixed before the threespine and blackspotted lineages diverged. The average read depth ratio in this stratum is 0.58. We defined a gene as hemizygous if the average read depth ratio across its coding region is less than 0.75 (White et al. 2015). By that measure, 92% of genes in stratum S1 have been lost from the Y (table 1).

We refer to the second oldest stratum on the Y as stratum R1 in order to distinguish it from strata on the threespine and Japan Sea Y chromosomes. Here, the extent of degeneration on the Y is similar to that seen in stratum S1: the average read depth ratio is 0.60 (fig. 3*A*), and 83% of genes are hemizygous (table 1). The next two strata, denoted R2 and R3, are less degenerate. Their average read depth ratios are 0.69 and 0.77, respectively, whereas 77% and 70% of their genes are hemizygous (table 1). Overall, we estimate that 85% (814/963) of the genes on the X have been wholly or partially deleted from the Y chromosome of the blackspotted stickleback. We find no evidence of increased accumulation of premature stop codons (49 genes on Y vs. 67 on X).

**Fig. 4.**
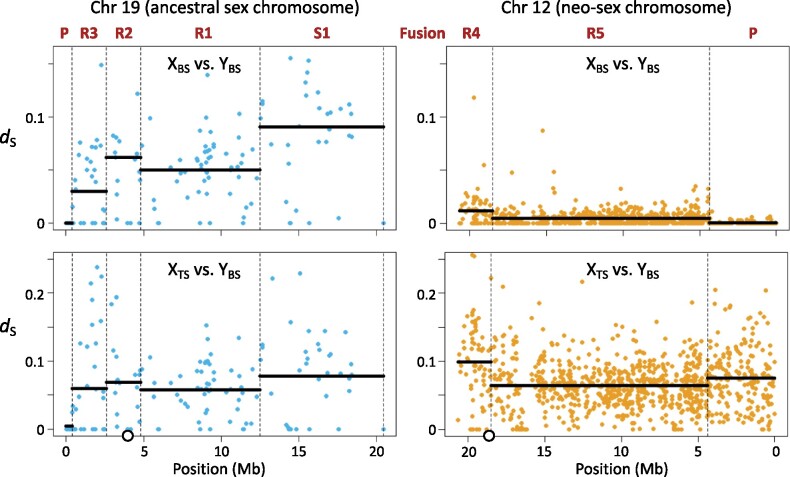
Divergence at synonymous sites (*d*_S_) for individual genes on Chr 19 and Chr 12. Top panels: *d*_S_ between blackspotted stickleback (BS) X and Y chromosomes. Bottom panels: *d*_S_ between blackspotted stickleback (BS) Y and threespine stickleback (TS) X chromosome (left) and Chr 12 (right). Dashed vertical lines represent boundaries between the PARs (labeled P) and the strata in the SDR (labeled S1 and R1 to R5). Solid horizontal lines show the mean for each stratum. The circles on the *X* axis of the lower panel show the locations of the centromeres on Chr 19 and Chr 12 in threespine stickleback.

Another pattern emerges, however, when we examine the overall read depth separately within the sexes (supplementary fig. S3, Supplementary Material online). In males, the read depth is consistently low across all four regions, whereas in females, it is substantially lower in strata R2 and R3 than in strata S1 and R1. This is surprising because the establishment of strata on the Y is not generally associated with degeneration on the X. We verified that the degeneration of the X in the SDR is not an artifact of our crossing design by sequencing four blackspotted females. Again, we found differences in read depth along the X but not on any autosome (data not shown), indicating that reads from this end of the blackspotted X are failing to map to the threespine stickleback reference genome. Why this occurs is unclear, as divergence statistics such as *d*_S_ are not elevated in this region (fig. 4). Mapping errors may result from increased accumulation of transposable elements in one of the stickleback species, as observed by Peichel et al. (2020) in the threespine stickleback PAR (which overlaps with the blackspotted stratum R3). The development of a reference genome for blackspotted stickleback could clarify the reason for this pattern.

The pattern of *F*_ST_ along the SDR of Chr 19 is also somewhat unexpected: it increases as one proceeds from the oldest stratum (S1) to the youngest (R3) (fig. 3*B*). The differences between all pairs of strata are significant (*P *<* *0.02, Mann–Whitney *U* test). The lack of a positive correlation between stratum age and sex chromosome differentiation has also been seen in other species with degenerate sex chromosomes, and is thought to result from bioinformatic artifacts (Vicoso and Bachtrog 2015). When a stratum is first established, *F*_ST_ initially increases as the X and Y began to diverge. But eventually the X and Y diverge so much that Y-linked reads fail to map to the X reference, resulting in hemizygous sites that SNP calling programs assume are homozygous, leading to the appearance of decreased differentiation (measured, e.g., by *F*_ST_ and *d*_S_).

Other measures shown in table 1 confirm that these regions are different strata. In particular, the rate of synonymous substitutions (*d*_S_) between the blackspotted X and Y at sites that are not hemizygous varies between the four strata on Chr 19, as expected if they stopped recombining at different times (table 1, fig. 3, and supplementary figs. S4 and S5, Supplementary Material online). We estimated the ages of strata R1, R2, and R3 by normalizing *d*_S_ between the blackspotted stickleback Y and X by the value of *d*_S_ between the blackspotted Y and threespine stickleback X (see Materials and Methods). Divergence between the blackspotted and threespine stickleback lineages is dated at 14 Ma (Varadharajan et al. 2019). Strata R1 and R2 stopped recombining soon after speciation, at 13 and 12 Ma respectively. Stratum R3 stopped recombining 7 Ma. Stratum S1 cannot be dated using this method, but Peichel et al. (2020) estimated that it evolved 22 Ma based on data from the threespine stickleback. The *d*_N_/*d*_S_ ratio is not significantly different between any of the strata (table 1 and supplementary fig. S4, Supplementary Material online).

### The SDR on the Neo-Sex Chromosome (Chr 12)

We now turn to Chr 12, which was an autosome that formed a neo-Y chromosome when it fused to the ancestral Y chromosome (Chr 19). This fusion doubled the size of the Y, as Chrs 12 and 19 are both approximately 20 Mb (fig. 1). Y monophyly clearly shows that the SDR has expanded across most of Chr 12, extending 16.4 Mb from the fusion (fig. 3*F*). Lack of X monophyly in most windows suggests that recombination was suppressed here recently, long after it was suppressed on Chr 19. The PAR makes up the remaining 4.4 Mb of the chromosome distal to the fusion, as shown by the lack of monophyly of either X or Y chromosomes (fig. 3*F*) and low values of *F*_ST_ between the neo-X and neo-Y (fig. 3*E*). Thus, the blackspotted sex chromosomes have two PARs, a small one on Chr 19 and much larger one on Chr 12.

The read depth ratio in the SDR of the neo-sex chromosomes is nearly equal to the autosomal mean, indicating that the neo-Y is young and has not degenerated much (fig. 3*D* and supplementary fig. S2, Supplementary Material online). Only 39 genes (2.5%) are hemizygous in the SDR of Chr 12. Although hemizygous genes occur along the entire SDR, they are mainly clustered in two regions. The first is proximal to the fusion (between 18.5 and 20.7 Mb) and includes 14 (36%) of the hemizygous genes. We tentatively identify this as a stratum and refer to it as R4. We refer to the remainder of the SDR on Chr 12 as stratum R5 (figs. 1 and 3). The second region that contains a high density of hemizygous genes (nine total) lies between 12.8 and 13.0 Mb. This region may represent a single deletion. Premature stop codons only occur in 1% more genes on the neo-Y than on the neo-X (73 genes on Y vs. 63 on X).

Additional evidence supports the existence of two strata in the SDR of the neo-Y on Chr 12. In total 9% of genes are hemizygous in stratum R4, but only 2% of genes are hemizygous in stratum R5. Stratum R4 also shows elevated *F*_ST_ (fig. 3*E*) and stronger X monophyly (fig. 3*F*), consistent with an older origin (*P *<* *10^−5^ for both statistics, Mann–Whitney *U* test). It has significantly greater *d*_S_ and *d*_N_ than stratum R5 (*P < *10^−8^, Mann–Whitney *U* test), but is not significantly different in *d*_N_*/d*_S_ (*P *=* *0.09) (table 1, fig. 4, and supplementary figs. S4 and S5, Supplementary Material online). Normalizing *d*_S_ by divergence with the threespine Chr 12 homolog (see above), we estimate that stratum R4 stopped recombining 1.7 Ma, and stratum R5 stopped recombining less than 1.0 Ma. These are likely overestimates because they represent the most recent common ancestor of all neo-X and neo-Y sequences, but the neo-X chromosomes are not monophyletic in many windows of the SDR (fig. 3*F*).

In sum, based on several statistics (the percent of hemizygous genes, *F*_ST_, *d*_S_, and *d*_N_) we find that there are two young strata (R4 and R5) on the neo-sex chromosome (Chr 12).

### The Y Chromosome Originated in the *Gasterosteus* Ancestor

The degrees of X–Y divergence and Y degeneration across Chr 19 are dramatically greater in the blackspotted stickleback than in the other two species of *Gasterosteus* (Ross and Peichel 2008; Leder et al. 2010; Natri et al. 2013; Roesti et al. 2013; Yoshida et al. 2014, 2017; Schultheiß et al. 2015; White et al. 2015; Dagilis 2019; Peichel et al. 2020). One explanation is that their Y chromosomes are not homologous, and the blackspotted Y is much older. We used two approaches to falsify that hypothesis. The first is based on a method that identifies shared duplications on the Y chromosomes, and the second uses multispecies gene trees. The results show clearly that the oldest stratum of the Y chromosome (S1 on Chr 19) evolved in the common ancestor of all three *Gasterosteus* species.

#### Shared Duplications on the Y Chromosome

Homology between Y chromosomes can be inferred from shared Y-specific duplications. Bissegger et al. (2020) discovered that at least 38 small autosomal regions have been duplicated onto the Y chromosome SDR in threespine stickleback. These autosomal regions appear to have extreme differentiation (*F*_ST_) between males and females, which is an artifact that results when sequencing reads from the duplicates on the Y chromosome mismap to their autosomal paralogs when using the female reference genome.

**Fig. 5.**
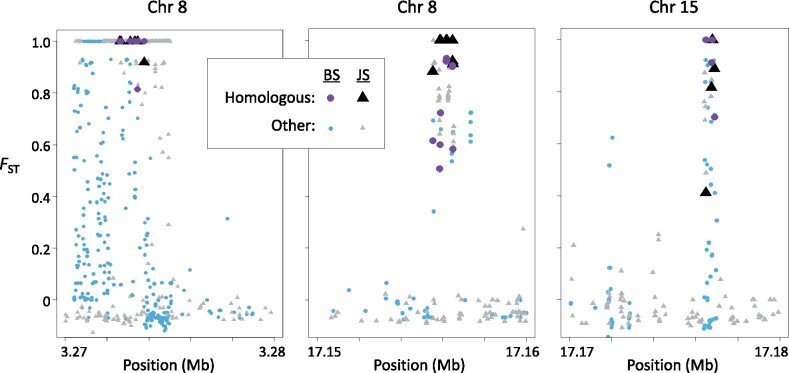
Three windows on autosomes include regions that duplicated onto the ancestral Y chromosome (Chr 19) in the common ancestor of *Gasterosteus* sticklebacks. *F*_ST_ between sons and daughters for alleles inherited from the father is shown for each SNP. Circles represent SNPs in blackspotted (BS) sticklebacks and triangles represent SNPs in Japan Sea (JS) sticklebacks. “Homologous” denotes SNPs that have *F*_ST_ >0.25 and a male-specific allele in both species, which provides strong evidence for Y chromosome homology. The window in the center panel contains the ortholog to the putative male-determining gene (*Amhy*) in threespine stickleback (Peichel et al. 2020).

Exploiting that discovery, we asked if some of these duplicates are shared between the three *Gasterosteus* species. We first calculated *F*_ST_ between the sons and daughters in our pedigrees for the autosomes that they inherited from their blackspotted father. Values were averaged over 10 kb windows. We repeated this analysis using comparable pedigrees from the Japan Sea stickleback studied by Dagilis (2019). For each of the two species, we located the windows whose *F*_ST_ values fall in the top 2% of the distribution. Of those, 183 windows are shared between blackspotted and Japan Sea sticklebacks. This number is far greater than expected by chance (*n *=* *14, *P *<* *10^−5^, χ^2^ test). In those windows, 98 SNPs are shared by both species. Three of these windows contain multiple SNPs where both species have high *F*_ST_ (>0.25) between the X and Y and the same male-specific allele (fig. 5). These SNPs, which we refer to as “homologous Y duplicates,” provide strong evidence that these three regions were duplicated from the autosomes to the Y in the ancestor of blackspotted and Japan Sea sticklebacks. Intriguingly, one of the windows (17.15 to 17.16 Mb on Chr 8) contains the ortholog to the putative male-determining gene (*Amhy*) in threespine stickleback, which arose by a duplication from Chr 8 to Chr 19 (Peichel et al. 2020). This finding suggests that the three *Gasterosteus* species share the same master sex-determining gene.

The homologous Y duplicates within these three windows show additional features consistent with autosome-to-Y duplications. The read depth ratio is consistently greater than one (supplementary fig. S6, Supplementary Material online), as expected if males have one or more Y duplicates in addition to the autosomal paralog. Reads containing the male-specific alleles for these SNPs typically comprise much less than half of the total reads mapping to the region. This pattern is expected when a mutation fixes in the Y paralog, since there are twice as many copies of the autosomal paralog in the genome. Finally, we find that the high *F*_ST_ regions in each of these three windows BLAST with high similarity to at least one region in the recently published threespine stickleback Y reference (Peichel et al. 2020), but not to any other region on the autosomes or X chromosome in the refence genome. All these data are strong evidence that these SNPs fall within regions that duplicated onto the Y of Chr 19 from autosomes in the common ancestor of *Gasterosteus* sticklebacks.

#### Multispecies Gene Trees

Different hypotheses for the origin of an SDR predict contrasting multispecies gene tree topologies (García-Moreno and Mindell 2000; Handley et al. 2004; Dixon et al. 2019; Natri et al. 2019). If the SDR on the Ys originated in the common ancestor of two species, then this region of their Y chromosomes will be more closely related to each other than to the X chromosomes of their own species (fig. 2*A*). Conversely, if the SDRs on the Ys arose from autosomes independently, then the SDRs of the X and Y within each species will be more closely related than they are to the SDRs of the other species (fig. 2*B*). Finally, if the SDR on the Y of one species originated from an X chromosome or the SDR (an X–Y turnover), then the new Y will form a clade with the X chromosome in that species, which will in turn be sister to the X chromosome from the other species (fig. 2*C*). Note that this use of gene trees differs from the Y monophyly analyses presented above, which use gene trees from multiple X and Y chromosomes of a single species to identify nonrecombining regions.

We determined the evolutionary history of the *Gasterosteus* sex chromosomes by constructing multispecies gene trees for nonoverlapping 100 kb windows. The sequence data come from two sets of pedigrees. We used four phased X and four phased Y chromosomes from the blackspotted stickleback fathers and eight phased X chromosomes from the threespine stickleback mothers that we sequenced in this study. We obtained four phased X and four phased Y chromosomes from Japan Sea stickleback fathers and eight X chromosomes from threespine stickleback mothers that were sequenced for Dagilis (2019). As an outgroup, we used Chr 19 from a ninespine stickleback that was computationally phased by Dixon et al. (2019).

**Fig. 6.**
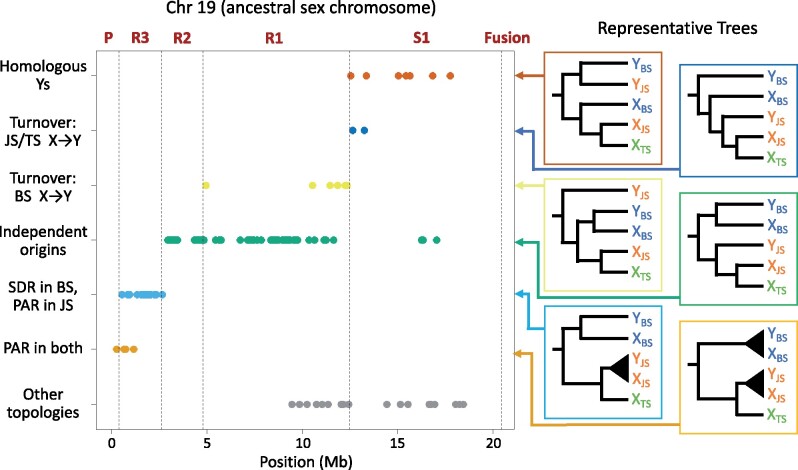
Multispecies gene tree topologies along Chr 19 reveal the strata have different evolutionary histories. Each dot represents the maximum likelihood topology for a 100 kb window. Representative trees associated with each topology are shown at right (BS, blackspotted; JS, Japan Sea; TS, threespine). “Other topologies” indicates topologies that do not correspond to a plausible evolutionary history, and likely result from genotyping and/or phasing error. Most windows in Stratum 1 have a topology indicating that the stratum arose in the shared *Gasterosteus* ancestor. Most windows in strata R1 and R2 have a topology consistent with strata that formed independently in blackspotted and in the ancestor of Japan Sea and threespine sticklebacks. Most windows in stratum R3 have a topology consistent with an SDR in blackspotted and a PAR in the ancestor of Japan Sea and threespine sticklebacks. Supplementary table 1, Supplementary Material online, shows how many genes trees were of each topology in each stratum.

In the most common topology seen in stratum S1 on Chr 19, the blackspotted Ys are sister to the Japan Sea Ys, and the blackspotted Xs are sister to the Japan Sea Xs (fig. 6; the numbers of gene trees that show each topology in each stratum are given in supplementary table 1, Supplementary Material online). This topology, which is not found in other regions, shows that this stratum evolved in the common ancestor of these two species and that neither species has experienced a sex chromosome turnover event since (compare with fig. 2*A*). This is strong confirmation that Chr 19 is the ancestral sex chromosome for the genus.

In strata R1 and R2, most windows exhibit a topology in which the blackspotted Xs and Ys are sister to one another, as are the Japan Sea Xs and Ys (fig. 6). This topology suggests the SDR expanded into these regions independently in the two species after they diverged (compare with fig. 2*B*). Stratum R3 lies in the SDR of the blackspotted stickleback, whereas it is in the PAR of the Japan Sea stickleback. As a result, the blackspotted Xs and Ys form clades that are sister to one another, whereas the Japan Sea Xs and Ys are intermingled (since they continue to recombine). Finally, the region from 0 to 400 kb features clades separating the species, but no differences between the Xs and Ys within species, consistent with it being a PAR in both species. Only 2% (2/93) of windows on Chr 19 show topologies consistent with an X-to-Y chromosome turnover, and these windows fall within regions that also feature several windows with biologically implausible topologies. Thus, these patterns likely reflect genotyping and phasing errors resulting from degeneration of the Y chromosomes in one or both species.

The neo-sex chromosome (Chr 12) is sex linked in blackspotted sticklebacks as well as the distantly related ninespine stickleback (*P. pungitius*), but not in the congeneric Japan Sea or threespine sticklebacks. Gene trees across the SDR of Chr 12 in the blackspotted stickleback confirm that its neo-X and neo-Y are closely related to one another, and that its neo-Y is young since its sequences are embedded with the neo-X sequences (supplementary fig. S7, Supplementary Material online). Thus, Chr 12 evolved independently as a sex chromosome in the blackspotted and ninespine sticklebacks.

## Discussion

Many studies have analyzed variation in the patterns and tempo of sex chromosome evolution. Most have compared species that share ancient sex chromosomes, for example among mammals and among birds (Hughes et al. 2005; Goto et al. 2009; Zhou et al. 2014; Xu, Auer, et al. 2019; Xu, Sin, et al. 2019). To date, genomic studies of young sex chromosomes have largely focused either on single species, on the sex chromosomes of related species that have nonhomologous Y or W chromosomes (e.g., Vicoso and Bachtrog 2013; Hough et al. 2014; Papadopulos et al. 2014; Crowson et al. 2017; Jeffries et al. 2018), and on species in which sex chromosome homology is ambiguous (Darolti et al. 2019; Kirkpatrick et al. 2020). Thus, the differences between species in patterns of X–Y differentiation and Y degeneration seen in these studies may reflect differences in the ages, identities, gene composition, or ancestral recombination landscapes of the sex chromosomes.

Clades sharing sex chromosomes descended from a relatively recent common ancestor offer unique insights into the repeatability of sex chromosome evolution, as they control for sex chromosome age and identity. *Gasterosteus* sticklebacks show exactly this situation. Using phased X and Y chromosomes, we obtained the first genomic picture of the blackspotted stickleback sex chromosomes and established their homology with the well-studied sex chromosomes of the threespine and Japan Sea sticklebacks. These data enable analyses (e.g., using gene trees and divergence between the X and Y) and insights (e.g., the ages and homologies of strata) not otherwise possible, and allow us to contrast the rates of evolutionary divergence among sex chromosomes.

### Contrasting Rates of Y Chromosome Degeneration in *Gasterosteus* Sticklebacks

We observed striking differences between the ancestral sex chromosomes (Chr 19) of the blackspotted stickleback and the threespine stickleback. Most notably, the SDR in the blackspotted stickleback is larger and more degenerate. Overall, 85% of genes have been deleted from the blackspotted Y, whereas only 56% have been lost from the threespine Y (Peichel et al. 2020). In stratum S1, which is shared between the species, the blackspotted is degenerating more quickly: only 8% of genes remain on the blackspotted Y, compared with 18% on the threespine Y (Peichel et al. 2020). The difference in the sizes of the SDRs arose because strata R1, R2, and R3 in blackspotted stickleback evolved independently from strata S2 and S3 in threespine and Japan Sea stickleback (fig. 1). The latter were established by chromosomal inversions that suppressed recombination between the X and Y (Ross and Peichel 2008; Peichel et al. 2020). We do not know if inversions were also responsible for the formation of strata in blackspotted stickleback. Stratum R4, for example, could have formed as the result of the fusion between the ancestral Y (Chr 19) and Chr 12 if the fusion and/or the nearby centromere inhibit crossovers between the X and Y (Manterola et al. 2009; Sardell et al. 2018).

The difference in degeneration of the blackspotted and threespine stickleback Ys reflects both the differing ages of their strata and differing rates of evolution. Strata R1 and R2 in blackspotted stickleback are much older (13 and 12 My) than strata S2 and S3 in threespine stickleback (6 and 4.7 My) (fig. 1), and it is therefore not surprising that they have lost more genes. In contrast, the age of stratum S1 is the same in both species as it evolved in their common ancestor. Nevertheless, it has lost 92% of its genes in blackspotted stickleback, but only 82% of its genes in threespine stickleback, indicating faster rates of Y degeneration in the blackspotted stickleback. Stratum R3 in the blackspotted stickleback, which was established 7 Ma, is similar in age to strata S2 and S3 in the threespine stickleback. Yet 70% of the genes have been lost from the Y in the blackspotted stratum R3, whereas only 24% and 31% have been deleted in threespine strata S2 and S3, respectively. The differences in rates of degeneration between nonhomologous, similarly aged strata could arise due to differences in gene content, but this is not true of the differences observed in stratum S1 (which the species share).

Why the ancestral Y (Chr 19) has degenerated much more rapidly in the blackspotted than in the threespine stickleback is not known. Degeneration of the Y chromosome can be accelerated by higher mutation rates, shorter generation times, and increased reproductive skew among males (Graves 2006), but there is no a priori reason to think the species differ in these ways. Perhaps the most likely hypothesis is that the blackspotted stickleback has experienced much stronger drift than the threespine stickleback. Observations supporting that idea are that its global range is much smaller (Wooton 1976), and it has much lower molecular diversity on autosomes (based on comparisons with Hohenlohe et al. 2010). Given the average molecular diversity on autosomes (π = 0.001) and assuming a mutation rate of 5×10^−8^, a naïve estimate of the effective population size based on θ (see Hahn 2019, Chap. 3), gives *N*_e_* *=* *5,000 for the blackspotted stickleback. This estimate is roughly 20% of the effective population size for threespine stickleback in the Pacific Ocean estimated by Ravinet et al. (2018). Strong drift therefore may have increased the fixation rate of slightly deleterious mutations on the blackspotted stickleback Y, including inversions that established the strata and fusion of the neo-Y. Another possibility is that conditions that create positive selection for the cessation of X–Y recombination could differ between the blackspotted and threespine lineages. Theory shows that stronger sexually antagonistic selection (in which different alleles are favored in females and males) and higher rates of recombination in the PAR can lead to the fixation of inversions on sex chromosomes that lead to the formation of strata (Rice 1987). (By suppressing recombination between a male-favorable allele and the SDR on the Y, for example, the inversion causes that allele to be permanently fixed on the Y.) Perhaps those conditions were met in the blackspotted lineage, leading to the earlier formation of strata that now extend over more of the ancestral Y.

### Conservation and Rearrangement of the Sex Chromosomes

The conservation of Chr 19 as a sex chromosome for at least 14 My in *Gasterosteus* sticklebacks contrasts with the high rates of sex chromosome turnover in other sticklebacks. Species in the genus *Pungitius*, for example, which have diverged over the last 4 My, vary both in which chromosome pair determines sex and in which sex is heterogametic (Ross et al. 2009; Dixon et al. 2019; Natri et al. 2019). Why sex chromosome turnover rates vary so much among groups of sticklebacks is a mystery. Perhaps some sort of constraint inhibits the origin of a new pair of sex chromosomes in *Gasterosteus* but not in other sticklebacks. One hypothesis is that sexually antagonistic selection, which can favor the maintenance of ancestral sex chromosomes (van Doorn and Kirkpatrick 2007), is stronger in *Gasterosteus*.

It is also unclear what evolutionary force fixed the fusion that created the neo-Y chromosome of the blackspotted stickleback. One possibility is the “fragile-Y” hypothesis (Blackmon et al. 2015). This theory posits that small PARs increase rates of aneuploidy in sperm because they provide little room for chiasma to form and ensure proper meiotic segregation. Fusions that expand the PAR may therefore be favored, and one pathway for that to happen is fusion of the ancestral sex chromosome with an autosome. Consistent with this idea, the PAR on the ancestral Chr 19 is very small (less than 500 kb), comprising less than 2% of the total length of the chromosome. Moreover, its small size likely predates the fusion with Chr 12, a precondition of the fragile-Y hypothesis. A second hypothesis is that sexually antagonistic selection favored the fusion (Charlesworth and Charlesworth 1980), as it may have done in Japan Sea stickleback (Dagilis 2019). The targets of selection must differ between the two species, however, since the fusions involve different autosomes. Third, the fusion may simply have drifted to fixation.

### Comparisons to Other Taxa

Only a handful of studies have reported differences between closely related species in the extent of recombination suppression, sex chromosome differentiation, and Y (or W) degeneration. The best examples come from birds. Although all birds share an homologous W chromosome, the number of strata, the degree of their degeneration, and size of the PAR vary widely between species that are distantly related (Zhou et al. 2014; Xu, Auer, et al. 2019; Xu, Sin, et al. 2019). There does not seem to be much variation in rates of degeneration between closely related birds (Xu, Auer, et al. 2019; Xu, Sin, et al. 2019), but their ancient W chromosomes are so highly degenerate (less than 10% of the genes are retained) that there may be little scope for further decay.

Other taxa do show variation in degeneration rate across homologous sex chromosomes. Hughes et al. (2005) observed much higher rates of degeneration on a part of the chimpanzee Y chromosome compared with its homologous region on the human Y. A small number of molecular markers in wild congeners of domesticated spinach suggest that rates of differentiation between homologous sex chromosomes differ (Fujito et al. 2015). Although the SDRs on the Y chromosomes of the plants *Rumex hastatulus* and *R. rothschildianus* are not homologous, they are of similar age, and yet they have degenerated to dramatically different degrees (Crowson et al. 2017). Darolti et al. (2019) reported extreme variation in sex chromosome degeneration between three closely related poeciliid fishes. However, our reanalysis of their data could not confirm that the highly degenerate Y chromosome of *Poecilia picta* is homologous to the undegenerated Ys of its congeners (Kirkpatrick et al. 2020). Thus, it is unclear whether rates of degeneration and evolution of X–Y differentiation since the origin of the Y chromosome actually vary in *Poecilia*. With the exception of the primate report, none of these studies were able to test whether rates of Y degeneration vary for homologous strata in different species, as we have shown in sticklebacks.

For this study, we developed a method to test for the homology of sex chromosomes between species by identifying shared autosome-to-Y duplications which manifest as *F*_ST_ peaks between paternally inherited “autosomal” loci in sons and daughters. This approach and a complementary method using multispecies gene trees are useful for comparing rates of sex chromosome differentiation and degeneration across closely related species. They also can be used to identify cryptic sex chromosome turnovers that involve the same linkage group. Fugu pufferfish and house flies (*Musca domestica*) are the only known cases of sex chromosome turnover events in which a new Y originated from an ancestral X chromosome (Meisel et al. 2017; Ieda et al. 2018). Such turnovers may in fact be quite common, however, as it is much easier to identify turnovers that involve a transition between different pairs of chromosomes or between XY and ZW sex determination. We suggest that comparative studies of sex chromosome evolution explicitly test for cryptic turnover and Y chromosome homology.

## Conclusions

Previous studies have documented several sex chromosome turnover events in stickleback fishes (Ross et al. 2009; Dixon et al. 2019; Natri et al. 2019). The results of this study show that the situation in sticklebacks is even more complex, as species in the genus *Gasterosteus* have sex chromosomes that differ substantially despite evolving from a common ancestral pair of sex chromosomes. Sex chromosomes in the blackspotted stickleback have degenerated much more rapidly than those in their congeners, perhaps because of a much smaller effective population size. Samples of phased young X and Y chromosomes can greatly advance our understanding of their evolutionary histories.

## Materials and Methods

### Sampling and Sequencing

All procedures involving live fish were approved by the Veterinary Service of the Department of Agriculture and Nature of the Canton of Bern (VTHa# BE4/16 and BE17/17) and the St. Mary’s University Animal Care Committee (17-18A2). All procedures involving live fish were approved by the Veterinary Service of the Department of Agriculture and Nature of the Canton of Bern (VTHa# BE4/16 and BE17/17) and the St. Mary’s University Animal Care Committee (17-18A2). During June and July 2017, we collected threespine stickleback (*G. aculeatus*) and blackspotted stickleback (*G. wheatlandi*) from Canal Lake, Nova Scotia, Canada (44.498654, −63.902952), blackspotted stickleback from York Harbour, Newfoundland, Canada (49.058555, −58.373138), and threespine stickleback from Humber Arm, Newfoundland, Canada (49.009842, −58.132643). Collections were carried out with permits for the Maritime Region (Licence 343930; FIN 700019217) and Newfoundland (Experimental Licence NL-4111-17).

We made 15 independent crosses by in vitro fertilization of the eggs of a threespine stickleback female with sperm from a blackspotted stickleback male. Five crosses were made using five blackspotted males and a single threespine female collected from Newfoundland, and ten crosses were made using ten blackspotted males and three threespine females collected from Nova Scotia. Interspecific crosses were used because they allow us to better phase the paternal and maternal X chromosomes in daughters. These interspecies F1 hybrid embryos start to develop but then arrest and never hatch (Hendry et al. 2009; Peichel C, personal observation). Crosses were raised until developmental arrest and then placed into 95% ethanol. DNA was extracted from fin clips of the parents and from individual embryos using phenol–chloroform extraction, followed by ethanol precipitation. The sex of the embryos was determined using microsatellite markers on chromosomes 12 (*Stn327*, *Pun2*) and 19 (*Stn284*, *Cyp19b*) that were previously shown to be sex-linked in blackspotted stickleback (Ross et al. 2009). For each of the 15 crosses, we sequenced the mother, the father, one son, and one daughter. DNA from each of these individuals was used to construct Illumina TruSeq DNA nano libraries, which were sequenced for 300 cycles (2×150 bp paired-end reads) in an S2 flow cell on an Illumina NovaSeq 6000. Library construction, sequencing, barcode trimming, and initial quality control was performed by the University of Bern Next Generation Sequencing Platform.

### Sequence Assembly and SNP Calling

We used *bwa mem* v.7.16 (Li and Durbin 2010) with default settings to map all raw reads to the most recent masked assembly of the threespine stickleback reference genome (Glazer et al. 2015). We sorted and removed all reads with low mapping quality (<20) using *SAMtools* v.1.6 (Li et al. 2009), based on the standard filtering threshold for this software. This resulted in 35X average sequencing coverage for autosomes. SNP calling was performed using the *samtools mpileup* and *bcftools call* functions in *SAMtools* v.1.6 (e.g., samtools mpileup -t DP, AD, SP -ugf glazer2015.unmasked.fa -r chrXIX *.minQ20.bam | bcftools call -f GQ -vmO z -o chrXIX.vcf.gz) (Li et al. 2009). We used *VCFtools* v.1.15 (Danecek et al. 2011) to filter the VCF file, retaining only biallelic SNPs with minimum quality scores of 999 (–min-alleles 2 –max-alleles 2 –remove-indels –minQ 999). Genotypes where the genotype quality was less than 20 were treated as missing data (–minGQ 20). Finally, we removed all SNPs where more than three sons or three daughters were missing data.

A fifth version of the assembly of the threespine stickleback genome was published at https://stickleback.genetics.uga.edu/, whereas this manuscript was in review (Nath et al. 2021). Importantly, it corrected numerous annotation errors in the earlier assembly that precluded accurate analyses of the coding regions. We used the new assembly to estimate divergence statistics (*d*_N_ and *d*_S_), the fraction of genes that are hemizygous, and the fraction of genes with premature stop codons. To do so, we generated a hard masked reference genome using the repeat annotations file with the *maskfasta* function in BEDtools v.2.26 (Quinlan and Hall 2010). We then used the pipeline described above to remap the sequence data from the crosses to the revised assembly with the threespine stickleback Y chromosome scaffold removed. Use of the revised assembly did not significantly change the results of other analyses.

We did not include the recent assembly of the threespine stickleback Y (Peichel et al. 2020) in our reference genome used for mapping for two reasons. First, we suspected and later confirmed that much of the SDR in blackspotted stickleback evolved independently from the same regions of the SDR in threespine stickleback. Therefore, we expected that some regions present on the blackspotted stickleback Y would be missing from the threespine stickleback Y reference, and vice versa. Use of separate X and Y reference scaffolds would result in chimeric mapping of the blackspotted stickleback Y reads, with some aligning to the X reference and others aligning to the Y reference. This situation would greatly complicate phasing and our ability to directly compare the X and Y sequences. Second, many of the analyses that we performed (including the use of male-to-female read depth ratio to quantify degeneration and the identification of homologous autosomal-to-Y duplications) rely on bioinformatic artifacts that arise when mapping data to a reference genome which lacks a Y scaffold.

### Phasing

We used a custom R script to phase the paternal and maternal gametes by transmission for parent–offspring trios, as described in Sardell et al. (2018) and Dagilis (2019) (see also supplementary fig. S1, Supplementary Material online). Briefly, for every heterozygous SNP in the offspring, we used parental genotypes to determine which allele was inherited from the father and which allele was inherited from the mother. Paternally inherited alleles in sons and daughters were transmitted in sperm containing a Y or X chromosome, respectively. This provided us with sequences of 15 blackspotted X chromosomes and 15 blackspotted Y chromosomes independently sampled from the wild. SNPs at which the offspring and both parents were heterozygous could not be unambiguously phased, so we conservatively treated them as missing data. Likewise, we removed any site where the offspring’s genotype included an allele that was not present in either parent. We then used a script provided by Dixon et al. (2019) to convert the genotypes into a haploid VCF file with a column for each gamete. We used custom phasing scripts rather than phase-by-transmission scripts from standard bioinformatic packages (e.g., GATK), because the latter are not specifically designed to account for transmission patterns in sex chromosomes and often produced clearly erroneous phasing results when applied to our data. Our approach is more conservative in assigning phased haplotypes to the X and Y. All custom scripts referred to in this section are publicly available, as outlined in the “Data Accessibility” section.

### Identification of SDR

We used *VCFtools –geno-depth* v.1.15 (Danecek et al. 2011) to extract the read depth for each son and daughter at each SNP. We then used a custom R script to calculate the ratio of the mean read depths in sons to the mean read depths in daughters (i.e., read depth ratio) in 10 kb windows.

Before further analyses, we filtered our genotypes using *VCFtools* v.1.15 to removes sites with excessive read depth, which likely represent duplications with multiple paralogs. We used 52 as the maximum mean read depth threshold, which represented 1.5 times the mean read depth on autosomes. We also removed sites where the mean read depth was below 26 (i.e., 0.75 times the mean autosomal read depth) to minimize genotyping errors at hemizygous sites. Finally, we used *VCFtools* v.1.15 to calculate weighted *F*_ST_ between the phased X and Y chromosomes. The maximum *F*_ST_ for all of our comparisons is 1 as we calculated differentiation between X and Y chromosomes rather than between males and females.

Gene trees within an SDR are expected to be “Y (or X) monophyletic” (Natri et al. 2013; Dixon et al. 2019; Toups et al. 2019). This condition holds if all of the Xs or Ys form a monophyletic clade with respect to the other sex chromosome. We used a custom R script to convert the haploid VCF files for each chromosome into fasta files comprising the SNP genotypes for each individual. We then used RAxML v.8.2.12 (Stamatakis 2014), employing the GTRGAMMA model and a rapid bootstrap analysis (*-f a*) over 1,000 bootstraps, to generate gene trees in 100 kb windows across Chromosomes 19 and 12. We calculated the fraction of Ys (or Xs) falling within the largest monophyletic clade of Ys (or Xs) using a custom R script that employs the R packages *ape* (Popescu et al. 2012) and *phytools* (Revell 2012). Gene trees are considered to be Y monophyletic when all Y sequences fall within a single monophyletic clade.

To quantify Y chromosome degeneration, we calculated the mean read depth ratio for each gene from data mapped to the fifth assembly of the threespine stickleback genome (Nath et al. 2021). Read depth for males and females was calculated using *VCFtools –geno-depth* v.1.15 (Danecek et al. 2011). Locations of genes were identified based on the Ensembl annotations (version 95) for this assembly. Genes where mean read depth ratio was <0.75 were considered hemizygous in accordance with White et al. (2015). We also identified all genes on the blackspotted X and Y that contained premature stop codons based on the consensus sequences described in the next paragraph.

We calculated genomic divergence using a pipeline and scripts developed by Dixon et al. (2019). This analysis used sequencing reads mapped to the fifth assembly of the threespine stickleback genome and the corresponding Ensembl annotations (Nath et al. 2021). First, we generated consensus sequences for the blackspotted X, blackspotted Y, and threespine X sequences from our pedigrees. We did this by subsetting the haploid phased vcf to include only the sequences for the group of interest. We also removed all SNPs where fewer than half the sequences contained the alternate allele using VCFtools with the flags “–min-alleles 2 –max-alleles 2 –non-ref-ac-any 1 –c.” This latter filter ensures that the consensus sequences include only the major alleles. We then used FastaAlternateReferenceMaker from the Genome Analysis Toolkit (DePristo et al. 2011) to generate the consensus sequences for each chromosome. Next, we used a custom script to generate individual sequences from the consensus for each gene annotated in the .gff file. We then removed all hemizygous genes based on the analysis described in the previous paragraph (mean read depth ratio <0.75). Finally, we used PAML v.4.9 (Yang 2007) to calculate *d*_S_, *d*_N_, and *d*_N_*/d*_S_ for each gene by comparing the blackspotted stickleback X and Y. We also calculated *d*_S_ by comparing the blackspotted stickleback Y to the threespine stickleback X. We estimated the age of each region by taking the ratio of the mean *d*_S_ between the blackspotted stickleback X and Y to the mean *d*_S_ between the threespine stickleback X and the blackspotted stickleback Y. We then multiplied the result by 14.3 My, which is the estimated age of the most recent common ancestor of the blackspotted and threespine sticklebacks (Varadharajan et al. 2019). We used the blackspotted Y in both comparisons to account for faster rates of Y chromosome evolution (Hughes et al. 2010). We removed genes with fewer than five SNPs when calculating *d*_N_*/d*_S_ on Chr 19 and fewer than eight SNPs on Chr 12, as these were the lowest thresholds that eliminated division by zero errors. Adjusting this threshold did not affect the results significantly.

We do not report results for molecular diversity (π) and Tajima’s *D* because the stringent filters required for accurately phasing the degenerate Y chromosome strongly bias those statistics. We established this by comparing the statistics from the phased data with those for unphased genome sequences we obtained from four adult blackspotted stickleback females from the same population. On the X chromosome, values of π estimated from the phased data ranged from two to five times lower than estimates from the unphased data. Values of Tajima’s *D* were similarly biased. We have no reason to think, however, that the other results reported here are also biased.

### Identification of Homologous Autosomal-Y Duplications

Our first approach for testing whether the sex chromosomes are homologous was to test whether chromosome duplications involving the Y are shared between species. Bissegger et al. (2020) noted that in threespine stickleback, many sites that map to autosomes in the Glazer et al. (2015) reference genome exhibit extreme differences in allele frequency between males and females. The observed allele frequency differences are biologically implausible as they require extreme mortality in the population (since autosomal allele frequencies will be approximately equal between males and females at conception). Bissegger et al. (2020) instead suggested that these regions have duplicated from the autosome onto the Y chromosome. Alternatively, they may represent regions on the divergent Y chromosome that have duplicated onto an autosome and do not have a similar paralog on the X. The important result is that Y-linked reads erroneously map to an autosomal region of the reference genome because the reference was obtained from a female and the X does not contain a homologous sequence.

We remapped the blackspotted stickleback sequences from this study to the unmasked assembly of the Glazer et al. (2015) threespine stickleback reference genome (since repeat-rich regions may be more likely to duplicate). We again applied the same filtering criteria as those used for initial identification of the SDR. We removed any SNPs where we had high-quality genotype data from fewer than eight sons or fewer than eight daughters. We did the same for a set of comparable phased Japan Sea X and Y sequences generated by Dagilis (2019). No similar data set of phased X and Y chromosomes exists for threespine stickleback.

We tested for homology between the blackspotted and Japan Sea stickleback Y chromosomes by identifying autosomal regions that show extreme differences in allele frequencies between males and females in both species. We first used *VCFtools* v.1.15 to calculate weighted *F*_ST_ between the phased X and Y sequences separately for each species in 10 kb nonoverlapping windows across all autosomes. We then used custom R scripts to identify windows in which mean *F*_ST_ falls within the top two percent of windows in both species. Within each of these shared outlier windows, we calculated *F*_ST_ between the X and Y sequences at each SNP, and identified all SNPs with *F*_ST_ >0.25 in both species. We further filtered the set of shared high-*F*_ST_ SNPs to include only those sites in which an allele is restricted to males in both species (as expected of mutations occurring on the Y paralog). Windows that contain multiple SNPs satisfying these criteria are interpreted as conclusive evidence that the Y chromosomes in blackspotted and Japan Sea sticklebacks are homologous. The alternative hypothesis of independent duplications would require that the same autosomal region was independently duplicated onto the Y multiple times, and also that it then independently accrued the same point mutations at multiple loci in less than 14 My.

To confirm that the putative Y duplications are also present in threespine sticklebacks, we used *blastn* v.2.8.1 with default settings to search for similar sequences in the threespine stickleback Y reference (Peichel et al. 2020) as well as the Glazer et al. (2015) threespine stickleback reference genome sequenced from a female. We also identified significant overlap between autosomal regions with high *F*_ST_ from our study and the putative autosome-to-Y duplications identified by Bissegger et al. (2020) for threespine stickleback. We do not present those results here, however, because the bioinformatic and analytic pipelines used in their study, including filtering criteria and measures of genetic differentiation, were quite different from ours, increasing the likelihood of type II errors.

### Multispecies Gene Trees for Identifying Sex Chromosome Homology

Our second approach for testing whether the sex chromosomes are homologous in all three *Gasterosteus* species uses multispecies gene trees. We included phased blackspotted stickleback X and Y sequences from four crosses in this study. Each cross used a different threespine stickleback mother, and we included the phased maternal X sequences in our analysis. Four phased X and four phased Y sequences from Japan Sea stickleback fathers, and their eight corresponding phased maternal threespine X sequences, were obtained from an earlier study that employed the same experimental cross design (Dagilis 2019). SNP-calling and phasing for the combined data set was done using the methods described above. Any sites with mean read depth less than 17 or less than 67 (representing 0.5× and 2× the mean read depth across all SNPs, respectively) were removed from the data set using *VCFtools* v.1.15 (Danecek et al. 2011). In addition, we modified our R phasing script to remove any sites within the SDR that were heterozygous in the father but where phasing indicated that brothers and sisters inherited the same paternal allele. Such inheritance patterns cannot occur within an SDR and likely represent genotyping or phasing errors arising from hemizygosity on the Y. We rooted the trees using a computationally phased *P. pungitius* genome from Dixon et al. (2019). Gene trees were constructed in RAxML v.8.2.12 (Stamatakis 2014) using the same parameters described above for testing XY consistency.

One potential problem with this approach is that genotyping errors in highly degenerate strata can lead to false topologies. For example, a SNP that is hemizygous in sons due to deletions on the Y will be assigned a homozygous genotype that incorrectly attributes the maternal threespine X allele to the blackspotted or Japan Sea Y. Therefore, we filtered the data set to only include windows where the maximum likelihood tree either features four reciprocally monophyletic clades representing the blackspotted Xs, blackspotted Ys, Japan Sea Xs, and Japan Sea Ys (as expected of old SDRs) or monophyletic clades for a species (as expected of PARs or new SDRs).

All windows within a nonrecombining stratum should share the same topology since they all descend from the same individual chromosome on which recombination was first suppressed (e.g., by an inversion). As described above, false topological inferences can arise from genotyping or phasing errors in regions of the chromosomes with large deletions on the Y. Therefore, we assume that the most common topology probably represents the true evolutionary history. Additionally, in our pedigrees, deletions on the Y are most likely to result in topologies in which one or both species’ Ys cluster with the threespine X sequences, since SNP-calling programs will wrongly impute the maternal threespine X allele as the blackspotted or Japan Sea Y genotype. Thus, topologies consistent with independent Y origins (fig. 2*B*) or Y turnover (fig. 2*C*) are far more likely to arise via error than topologies consistent with a single Y origin (fig. 2*A*). 

## Supplementary Material

Supplementary data are available at *Molecular Biology and Evolution* online.

## Supplementary Material

msab179_Supplementary_Data
