# Supplementary material for: Heterogeneous Histories of Recombination Suppression on Stickleback Sex Chromosomes
Source: Mol Biol Evol. 2021 Jun 12;38(10):4403–18. doi: 10.1093/molbev/msab179 (PMC8476171; doi:10.1093/molbev/msab179)
Supplement: msab179_Supplementary_Data [file msab179_supplementary_data.zip › Replies to reviews.docx]

Replies to the third set of reviews of MBE 21-0119

May, 2021

We are pleased to submit this, the fourth, version of our manuscript. Hundreds of changes have been made, and quite a few of those are substantial. Highlights include:

• A new Fig. 1 that summarizes the physical structure and evolutionary histories of the sex chromosomes in the blackspotted stickleback and its two congeners. We hope this will go a long way towards averting confusion about what is an admittedly complex evolutionary story.

• A new Fig. 2 and text at lines 102-104 clarify our use of the term “homologous”, which caused confusion in previous reviews.

• Much stronger statistical support is given for the existence of two distinct strata, R4 and R5, on the neo-sex chromosome (Chr 12) (lines 256-261). This point was controversial to previous reviewers.

Based on Heather Rowe’s email of April 15, our understanding is that this revision will not be sent back to Reviewers 1 or 2. It may or may not be sent back to Reviewer 3. For completeness, we include here our responses to the other two reviews as well.

**Review 3**

3.1) First, please note the confusion regarding the uploaded versions: “Sardell_2020_MBE_2Feb2021_revision_markup.docx” overwrites the text of “MBE-21-0119_Proof_hi.pdf”. This made me believe that you had mistakenly uploaded a previous version of the paper as the pdf-version. I understand that this was not the case: in the email dated 4th March, it is clarified that the old version of Fig 1 shows a phylogeny (which corresponds to the legend of Fig 1 in the markup-version), whereas the new version of Fig 1 shows the organization of the strata (which corresponds to Fig 1 in the pdf-version). How and why the markup-version ended up overwriting the current version remains a mystery to me…

This is strange, and we apologize for the confusion. Perhaps the overwrite occurred because we were asked to submit this version as a new manuscript, and we also included a markup version from the previous round of reviews?

3.2) This paper of sex chromosome evolution and recombination suppression in the blackspotted stickleback is, I believe, of interest in particular because the presence of a Y-autosome fusion (chr19+chr12), and because the results can be related to previous data from the close relatives, threespine stickleback and the Japanese sea stickleback. The main results include discovery of 6 putative evolutionary strata, estimates of the timing of these suppression events, and a higher rate of degeneration on the blackspotted Y compared to the same chromosome in other species. A main conclusion is that homologous sex chromosomes can evolve quite differently between species over short evolutionary time scales, which confirms results from other study systems (e.g. in tinamous, paleognaths). Additional strengths include (i) using controlled crosses allowing phasing of genomic sequences to separate X and Y chromosomes, (ii) rigorous bioinformatics analyses in general, and (iii) confirming that the related species share the same ancestral Y chromosome (or at least the part corresponding to stratum 1).
 Weaknesses/major comments: The most pressing issue is the unexpected finding that read coverage depth variation in females seems to be driving the male-to-female read depth ratio along the sex chromosome, and not, as would have been expected, variation in male coverage due to Y chromosome regions of different age and degeneration. The authors discuss this only very briefly and without a convincing explanation, despite the fact that this suggests that some of the putative strata (2-4) may have been defined by variation along the X rather than by Y variation. I suggest that the authors investigate this further as it affects the whole context of the study, which is written in light of what we expect, i.e. rapid evolution of Y.

We first would like to thank the reviewer for their thoughtful and constructive feedback on our work. As they point out, the read-depth variation in females among strata was surprising. Using additional unpublished whole genome sequences from adult female blackspotted sticklebacks, we again observed variation in mapping rates along Chr 19 but no other chromosomes. The variation is therefore real and not an artifact of our cross design. Why this occurs is unclear. As we discuss at lines 213-215, it is possible that this may result from increased accumulation of transposable elements in one of the stickleback species, as was observed by Peichel, et al. (2020) in the threespine stickleback PAR which overlaps R3.

The future development of a high-quality reference genome for the blackspotted stickleback likely will shed insight into this phenomenon, but this is not yet available for us to investigate in more detail.

Nonetheless, a key point is that read depth is only affected in what we now call strata 2 and 3, and read-depth is only one of several statistics that support the existence of different strata. Downstream filtering by mean read depth removes loci affected by X mapping issues from our analyses that rely on *d*_S_ or gene trees, which also point to the existence of different strata.

3.3) Furthermore, the motivation for defining two strata (5 and 6) on the fused regions (chr12) is weak (required some quite unstandardized definitions).

What we now call strata R4 and R5 differ significantly in the degree of sex chromosome differentiation (Fst), divergence at synonymous and at nonsynonymous sites, gene loss, and X-Y monophyly. We greatly strengthened the statistical evidence at lines 258-261, Table 1, Fig. 3, and Supp. Fig. S7.

3.4) I did not think it was entirely justified to use the epithet “new” for the method of using shared insertions to confirm that today’s Y chromosome (stratum 1) is of shared origin in threespine and blackspotted. The other method they are using, the phylogenetic method, also uses information of shared Y-polymorphism between species (SNP variation) to conclude that recombination between X and Y took place before the blackspotted split from threespine. So rather than using a new method, you have used a unique kind of mutation (translocations) to support the timing of recombination suppression.

We changed “new method” simply to “method” at lines 26 and 275. However, we do highlight the utility of this approach at lines 469-474 in the hopes that this will be a useful method for others.

Specific comments by lines: 

3.5) Lines 44-48: I believe that one should be careful with promising too much of a specific study system. It is here written that “Stickleback fishes (family Gasterosteidae) present an opportunity to test these questions [referring to the several questions listed in the previous paragraph], as they show extraordinary variation in sex determination.” As a reader one wonders whether sticklebacks are diverse enough to test all these questions with some degree of power? Some additional information (e.g. number of species, number of transitions, etc.) would have been valuable.

This is a good point. We removed the term “extraordinary”, and have clarified the questions we hope to answer with sticklebacks at lines 51-55. Key facts (such as the number of transitions) have not yet been determined, and the taxonomic status of some groups within the *Pungitius* genus is not clear, making it difficult to provide species numbers. We give citations to the literature that represents the current state of knowledge.

3.6) Lines 73-75. I agree that the present genome-wide study takes a big step forward in terms of details and understanding, but it also remains true that Ross et al.’s work did already suggest that chr19 is highly degenerated and chr12 less so. Of course, there is great value in the present data, but still, some of the main conclusions are complementary rather than conceptually novel.

We agree, and we cite Ross et al. appropriately (and note that Peichel is an author on that paper and this one). But Ross et al. was based on cytogenetics and genetic linkage mapping with microsatellites. It did not assess the extent of sequence degeneration on either the neo-sex chromosome (Chr 12) or the ancestral sex chromosome (Chr 19), identify the strata on Chr 12 or 19, the homology of the Chr 19 strata in the threespine and blackspotted stickleback, or the timing of degeneration. We provide examples of questions that we addressed with genomic data at lines 91-95.

3.7) Lines 82-86: I think it would have been preferable to refer to the Y chromosomes as being of shared origin within Gasterosteus rather than using the term homology as the latter is sometimes used in a broader context to indicate of shared origin (e.g. after a whole genome duplication event the two copies of a chromosome are homologous, and one may even define X and Y as homologous). Also, it is “only” stratum 1 that is “homologous”, and in fact within blackspotted the closest homolog to Y-linked genes on the other strata are their X equivalents.

This is a semantic issue, but it is important. We acknowledge that “homologous” is used in different ways by different workers. We have clarified our definition and emphasize that it differs from how some other workers use the term in the paragraph starting at lines 100-104.

3.8) Lines 97-105: SDR. A non-recombining region does not need to be sex-determining. Better to use non-recombining region or recombination suppressed region, or similar.

“SDR” (sex determining region) is a term of art: it is the standard terminology used throughout the field for a non-recombining region that contains the sex determining gene, as is the case in blackspotted stickleback. We strongly prefer to use terms like this that are embedded in the sex chromosome literature.

3.9) Lines 109-136: This section of the Results is actually methods and discussion – can be shortened substantially.

While we agree that there is a bit more ‘methods/discussion’ than normal, we include a broad discussion of methods at the beginning of the Results section because we believe they provide important context for interpreting our results. This is the first time that multiple samples of experimentally phased sex chromosomes have been studied. Readers will likely not be familiar with the approach, and indeed it was a source of confusion with another reviewer. Since the methods are placed at the end of the manuscript in MBE, we worry that many readers who read the sections sequentially will be confused if we do not include this material here. For these reasons, we have chosen to keep this section as written.

3.10) Lines 117-119: I agree, it was a good decision to not include the recently published threespine Y reference.

Thank you for recognizing the validity of this decision, which has caused contention in prior rounds of reviews.

3.11) Line 139: Here you point out “the nonrecombining SDR”. Does this mean that there also is a recombining SDR? See comment lines 97-105.

“Nonrecombining SDR” was redundant, and we have deleted “nonrecombining”.

3.12) Line 140: “a read depth ratio” spell out male-to-female read depth ratio…

We defined “read depth ratio” as short-hand for “male-to-female read depth ratio” on line 126. We prefer the abbreviated form as it appears 24 times in the manuscript

3.13) Line 154-155: Regarding “Windows with incomplete Y monophyly might also result from rare gene conversion between the X and Y.” This is actually a critical statement, because if gene conversion occurs it would substantially make inferences of shared origin (their homology) difficult. I suggest including a test of gene conversion, or a statement why this is not possible.

Our study, like studies of sex chromosomes before it, is limited to describing evolution of crossover recombination suppression. We agree that it would be very interesting to estimate the extent of gene conversion between the X and Y, but have been stymied when we attempted to do so in other stickleback species. To date, gene conversion between the X and Y has only been demonstrated in humans, where it is believed to be rare and remains poorly understood (Trombetta, et al. 2017). Therefore, we suspect that inconsistent gene trees likely reflect mapping or phasing errors in highly degenerate regions. We changed the sentence to emphasize the rarity of gene conversion events (lines 181-183).

Gene conversion cannot produce topologies that indicate a shared origin of a stratum between two species, as we show for Stratum 1. Further, our analysis that identifies shared duplicates from the autosomes (starting at line 280) is unaffected by gene conversion and provides independent support that the blackspotted and threespine stickleback Y chromosomes are homologous.

3.14) Line 197: Edit: suggests.

Fixed.

3.15) Lines 218-220. Regarding “These are likely overestimates because they represent the most recent common ancestor of all neo-X and neo-Y sequences, but the neo-X chromosomes are not monophyletic in many windows of the SDR (Fig. 2F).” Here a suggestion would be to compare one pair of sequences at a time to get a range of estimates that can then be compared to the much lower estimate of 10^4 years suggested above. The discrepancy between the two given suggestions 10^7 and 10^4 needs to be addressed.

We thank the reviewer for pointing out this discrepancy. We deleted the lower estimate (10^4^ years) because that was a *very* rough lower bound based on neutral coalescent theory. The estimates now based on *d*_S_ (at line 261) are expected to be much more accurate.

3.16) Line 284: I think it is important here to point out whether the branches were significantly supported or not. The trees for single genes/windows can even be given as supplementary material with bootstrap support added for the different branches.

There are hundreds of gene trees, so it is not practical to show bootstrap support for each tree. But we have found a solution that responds to the spirit of this suggestion. The new Supplementary Table 1 show the number of gene trees with each topology within each stratum. We think this gives readers all the data needed to evaluate our conclusions critically.

3.17) Results: Very few statistical tests are performed with p-values given only for some possible comparisons, at lines 215-216, 245 and Table 1.

We have strengthened the statistical reporting. We now state that the differences that are significant between the strata in *d*_S_, *d*_N_, *F*_ST_, and monophyly at lines 219, 258-260, and Table 1.

**Reviewer 1**

1.1) This study is described a bit more clearly than previously. The study describes phased X and Y chromosome sequences and corirms that the oldest stratum of the Gasterosteus sex chromosomes (Chromosome 19) is present in the ancestor of this species and in threespines (G. aculeatus and G. nipponicus). The most interesting conclusion from the results is that recombination has been suppressed repeatedly in a modest evolutionary time. However, there may be some doubts about how many events occurred (see below), and there are still a number of things that are not at all clearly described or explained.

Regarding the importance of our results, we note that a review by D. Charlesworth (2021, *Evolution*) concludes that “there is a need for more quantitative approaches to studying sex‐linked regions”, and “describe[s] a scheme to help understand the relationships between different properties of sex‐linked regions … focus[ing] on their sizes (differentiating between small regions and extensive fully sex‐linked ones), the times when they evolved, and their differentiation.” Our paper follows the approach outlined in that article and presents some of the first quantitative results that Charlesworth says are lacking in the field.

1.2) A first conclusion that is clear is that after the blackspotted lineage diverged from the threespines, a new stratum of suppressed recombination evolved on chromosome 19 (showing clear X-Y differentiation and Y degeneration). The conclusion that the younger chromosome 19 strata in the blackspotted species evolved independently of those in the threespine needs to be made clearer.

This result is supported by several independent analyses, including dating from divergence statistics (Table 1) and gene trees (Fig. 6). The conclusion that the younger strata evolved independently in threespine and blackspotted sticklebacks is shown clearly in Fig. 1 and is stated throughout the manuscript (e.g., lines 29, 123, 142, 340, 383). To further clarify this conclusion, we followed Reviewer 2’s suggestion and combined Fig. 1 and Fig. 7 into a new Figure 1. It provides a clear summary of the sex chromosome structures and timing of events that will help readers to follow the manuscript.

1.3) Ideally, readers could be able to compare the extents of the of the genome regions in both the species (Figure 2 shows only the latter, and Figure 1 is not detailed enough to show the differences clearly, and is potentially confusing, because the claimed S2 presumably evolved independently in the 2 species, and should perhaps not be given the same name).

The data presented in Fig. 2 (now Fig. 3) are for the sex chromosomes of the blackspotted stickleback, which have not been previously analyzed on the genomic level. Several other studies have summarized the structure of the sex chromosomes in threespine and Japan Sea sticklebacks. We do not feel that it is appropriate for this manuscript to act as an exhaustive review of the results of the many previous studies of the sex chromosomes in other species of sticklebacks. Instead, we have provided a general overview of the most relevant data on the threespine and Japan Sea stickleback sex chromosomes in the introduction (lines 56-62) and revised Fig. 1 to summarize this information.

We do agree with the suggestion to give different names to strata that evolved independently in different species. The strata specific to the blackspotted stickleback have been renamed R1 to R5.

1.4) Even if one does not accept that the threespine evolved 2 younger strata (and simply views them as a single one, see below), …

We do not understand why Reviewer 1 believes that threespine sticklebacks only evolved a single younger stratum. Peichel et al. 2020 conclusively demonstrated that the threespine stickleback SDR comprises 3 strata of different ages that correspond to known inversions (noted on lines 71-73 and 384-386).

1.5) … and similarly for the blackspotted species, clear evidence that they evolved independently in these 2 species would be interesting. If the region in the blackspotted species is bigger, or older (or both) it would be unsurprising that it might be more highly degenerated). At present, these straightforward bits of information are not clearly shown.

As noted above, we present independent analyses (multispecies gene trees (Fig. 6) and dating using divergence statistics (Table 1)) that show the new strata evolved independently in blackspotted and threespine/Japan Sea lineages. The sizes and ages of the strata in blackspotted stickleback are provided in Table 1 as well as the text, and the sizes and ages of the strata in threespine stickleback are shown in the new Figure 1. We also clearly compare their ages and extent of degeneration in the second paragraph of the Discussion section “Contrasting rates of Y chromosome degeneration in *Gasterosteus* sticklebacks” (line 376).

1.6) The statement that “suppressed recombination evolved much more rapidly in blackspotted stickleback” is also unclear. Presumably it means that it evolved sooner after the shared oldest stratum arose (and the threespine underwent a parallel change after a longer delay)?

With respect, we do not see any ambiguity in our statement. After the blackspotted and threespine lineages diverged, regions of suppressed recombination evolved more quickly on the blackspotted Y than the threespine/Japan Sea Y: see Figure 1.

1.7) But it is not clear what is meant by “differing rates of evolution”.

This phrase only occurs in the sentence, “The difference in degeneration patterns between the Y chromosomes of blackspotted and threespine sticklebacks reflects both the relative ages of their strata and differing rates of evolution” (lines 390-391). That sentence was added in response to prior suggestions from Reviewer 1. We believe it is difficult to misinterpret: Y degeneration evolved more rapidly in blackspotted stickleback over the same time scales. We clearly explain the evidence for this finding in the rest of that paragraph.

1.8) The study also documents a recent fusion between the ancestral blackspotted stickleback Y chromosome and an autosome (Chromosome 12), producing neo-X and neo-Y regions. I think that these have not undergone X-Y differentiation and Y degeneration.

This statement is simply wrong, and it calls into question how closely Reviewer 1 read our manuscript. Fig. 3E clearly shows extensive X-Y differentiation (*F*_ST_) across the entire SDR of Chr 12. Data on gene loss also indicate that the neo-Y has degenerated (lines 249-252, Table 1)

1.9) Together, the results are claimed to identify 6 strata, but the text does not make the conclusions as clear as they might be. This high number is surprising, and needs to be evaluated carefully. First, these are not clearly stated in the text. I think that, of the 6 claimed strata, 4 are claimed on the ancestral XY pair (19) and 2 in the neo-sex chromosome (12) region (it would be helpful if the numbers were mentioned clearly in the text, with a pointer to readers that this can be seen in Figure 2; Figure 7 also caused me confusion, because there is looks as if S4 is also in chromosome 12).

The strata are very clearly identified in the text and figures. We note the presence of six strata on line 34 of the abstract, consistently refer to the strata by number. We have added Figure 1 which clearly shows their location and ages.

1.10) Second, the conclusion is based on Y-X divergence results.

That statement is wrong: the strata are not identified based on X-Y divergence. As is clearly stated at lines 184-187, “We identified the boundaries of four putative strata using two methods: an algorithm that detects changepoints in the read depth ratio data (Killick and Eckley 2014) and multispecies gene trees (described in a later section)”.

In addition to Y-X divergence (*F*_ST_), the strata are significantly different in We now state that the differences that are significant between the strata in *d*_S_, *d*_N_, and monophyly (lines 219, 258-260, and Table 1). These statistics have been used to define strata in many previous studies, including those of the threespine stickleback (e.g., Lahn and Page 1999, Handley *et al.* 2004, Roesti et al. 2013, Papadopulos *et al.* 2015, Wright *et al.* 2017, Peichel *et al.* 2020, Almeida *et al.* 2020).

1.11) Second, confidence intervals are not given for the values, so it is unclear how much of the variability in Figure 1 is due to short sequences with few synonymous sites, and whether some quality control in terms of lengths might be helpful.

We have added Supplementary fig. S5 with violin plots showing the distribution of *d*_S_ and *d*_N_ for genes within each stratum. However, these calculations are all based on annotated genes, with between 78 and 1379 genes per stratum (Table 1). It is difficult to imagine that these results are the result of short sequences with few synonymous sites.

1.12) Third, the values differ very little between some of the claimed strata (and the values don’t even increase monotonically with distance from the PAR), and the values themselves are small for in S5 and 6, so one might be concerned that the possibility of other differences was not considered.

The reviewer is incorrect once again. As described above, strata were first identified using a changepoint algorithm which detects significant differences in trend data. This approach is much more scientifically rigorous than the “eyeball” test employed by this reviewer. As outlined in the caption to Table 1, we found significant differences in *d*_S_ between strata except comparisons involving stratum R2, which retains relatively few genes on the Y (see the table caption). Differences in *d*_S_ between R4 and R5 are highly significant (line 259-260 and Table 1 caption).

Reviewer 1 does correctly note that the values of *d*_S_ don’t monotonically increase with distance. We have expanded our explanation of that finding at line 219-224: “The lack of a correlation between stratum age and sex chromosome differentiation has also been seen in other species with degenerate sex chromosomes, and is thought to result from bioinformatic artifacts (Vicoso and Bachtrog 2015). When a stratum is first established, *F*_ST_ initially increases as the X and Y began to diverge. But eventually the X and Y diverge so much that Y-linked reads fail to map to the X reference, resulting in hemizygous sites that SNP calling programs assume are homozygous, leading to decreased differentiation (including *F*_ST_ and *d*_S_).”

1.13) Differences in mutation rates should be taken into account by using divergence from a suitable outgroup species. In this case, the ideal data are available — the orthologs of the threespine (and Fig. 3 shows both kinds of data). Indeed, from Table 1, it looks as though higher Y-X dS values correlate with higher threespine X-wheatlandi-Y values., and the synonymous site Y-X divergence values in wheatlandi, relative to the inter-species values, range from 0.5 to > 1 for the oldest one (and the strata in the threespine have the same rank order of relative dS, with values from 0.48 to 1.99). The strata with similar dS values may therefore not actually represent different recombination suppression events, but could reflect different sets of genes, or different genomic regions, with different mutation rates. This should be properly investigated, including asking whether sequences in the oldest stratum, with the highest relative dS values, are as well aligned as those in the younger one, as this is another possibility for the results.

These concerns are unfounded. We controlled for differences in mutation rates across genes when estimating ages of strata. That is, we scale *d*_S_ values between the blackspotted stickleback Y and X by the *d*_S_ values between the blackspotted stickleback Y and the threespine stickleback Y (as explained in lines 228-230).

1.14) The wheatlandi sequence reads were mapped to the repeat-masked version of the threespine stickleback female reference genome, and this is another potential source of problems.

We followed the standard bioinformatic protocol for analyzing whole genome sequences. Using repeat-masked versions of reference genomes is universally agreed to be the appropriate methodology because repeat-rich regions introduce mapping errors.

1.15) Overall, I am not 100% convinced that new strata have evolved on chromosome 12, especially given the very slight degeneration estimates. The main evidence is the very high Fst. Oddly, the low Fst values seen in the older chromosome 19 strata are absent here. Have the authors looked at linkage disequilibrium in a sample from a natural population? If this chromosome really shows complete Y linkage across the roughly 12 Mb region, it should be clear in an analysis of LD.

Again, we present several lines of evidence for strata on chromosome 12: number of hemizygous genes, *d*_S_, Fst, and X-Y monophyly (Fig. 3 and Table 1). The low Fst values seen on chromosome 19 result from the well-known effect where observed X-Y differentiation is reduced due to bioinformatic artifacts in regions where the Y is hemizygous (see Vicoso and Bachtrog 2015). This is explained at lines 219-224.

In effect, we have already demonstrated high LD across this region, and then gone further. The multispecies gene trees (Fig. 6) cluster chromosomes with similar alleles, which is to say they are in high LD. The gene trees can be viewed as high-dimensional representations of linkage disequilibrium. The evidence for extreme disequilibrium, and therefore the absence of recombination, is unequivocal.

1.16) Fourth, if the dS value for the oldest stratum, which is shared, is trustworthy, one would expect them to be similar in the threespine and wheatlandi, but the values in Table 1 they differ considerably from those in the recent paper about the threespine (Peichel et al., 2020 Genome Biology 21: 177. doi: 10.1186/s13059-020-02097-x; the values are 15.5% versus 8%, respectively). I don’t understand this big difference in the shared oldest stratum.

This result is a major conclusion of our paper and has been discussed above. Our data show unambiguously that degeneration evolved much more rapidly in blackspotted stickleback than in threespine stickleback, as reflected in the different values of *d*_N_ and gene loss for Stratum 1! Reviewer 1 seems to prefer to ignore the evidence that we present merely because it does not agree with their *a priori* assumptions about the system.

The title and focus of the original submission of this manuscript was on the differing rates of degeneration in the Y chromosomes of the blackspotted and threespine lineages. But a different reviewer objected that this result was not novel, correctly noting that several previous papers have demonstrated the fallacy of the “clock” view of sex chromosome evolution. We therefore extensively rewrote the manuscript. Here Reviewer 1 claims that our results must be wrong because they are inconsistent with the expectation that degeneration evolves via a predictable clock-like fashion. Our data (and others) show that view is incorrect.

1.17) Overall, a much clearer comparison with the threespine results would be very helpful, probably in Table 1.

Table 1 presents the original results of our study. Several previous studies cited in our manuscript characterized the sex chromosomes of threespine and Japan Sea sticklebacks, and we contrast those results to ours in the Discussion in the section titled “Contrasting rates of Y chromosome degeneration in *Gasterosteus* sticklebacks” (line 376). The new Figure 1 greatly facilitates comparisons between species.

1.18) Similarly, the extent of degeneration differs a lot (18% of genes remaining in threespine, versus only 8 or 9% in the blackspotted). I think that some explanation should be given for the much greater differentiation in the threespine, which is the basis for the statement in this ms that “the Y degenerated more rapidly in the blackspotted stickleback”.

At lines 31 and 405 we suggest that the much smaller effective population of blackspotted stickleback is most likely responsible.

Reviewer 1 is incorrect when they say that “the much greater differentiation … is the basis for the statement … that the Y degenerated more rapidly.” The conclusion that the blackspotted Y degenerated more quickly is based primarily on the genes lost. This reviewer requested that analysis in the last round of reviews, and that is exactly what we have done.

1.19) The oldest (shared) stratum is presumably the only one where rates can be compared (for the other strata, different times when recombination stopped, are confounded with different sizes of the regions, which will also carry different sets of genes, which might degenerate at different rates).

We agree that the shared Stratum 1 offers the best evidence that rates of Y degeneration vary between species (lines 380-382 and 394-395). We disagree, however, that major differences in rates of degeneration between non-homologous strata on the same sex chromosome are not noteworthy. Strata of similar age in the two species exhibit substantial differences in gene loss (lines 396-399). We have added a sentence to the discussion noting the concerns of Reviewer 1 that differences in gene content may partially explain differences in degeneration rates (lines 399-401).

1.20) As written, the text about rates is quite confusing (“the ancestral Y (Chr 19) is degenerating much more rapidly in the blackspotted than in the threespine stickleback”  fails to make clear enough that it is just the shared Y-linked region of chr 19, and this needs to be discussed clearly.

Again, this is not true. As explained in the reply to the previous comment, strata of similar ages also show strong differences between the species in Y degeneration rates. There is no evidence that the faster rates of degeneration are restricted to Stratum 1, as implied by Reviewer 1.

1.21) The authors conclude that the wheatlandi lineage experienced a higher rate of Y degeneration (in the older stratum) than in the other species, and suggest that this may be explained by a much smaller effective population size in the blackspotted stickleback. Is there any evidence for a low Ne? What is the level of diversity? Is there any evidence for a recent bottleneck (if the time is appropriate for testing this).? Could a difference be big enough to explain the magnitude of the difference? Are non-synonymous substitutions accelerated in this lineage, in sequences that are still present on the Y?

The data that the reviewer requests were already given at lines At line 405, where we provide evidence for a lower *N_E_* in blackspotted stickleback, including estimates of molecular diversity. The values of *d*_N_ follow the same patterns as those for *d_S_.*

1.22) … may have been favored by the very small size of the recombining region on the ancestral sex chromosome. We identify six strata on the ancestral and neo-sex chromosomes where recombination between the X and Y ceased at different times. 
These results confirm that homologous sex chromosomes can evolve large differences even over short evolutionary timescales.

These sentences are excerpts from our abstract with no comment from Reviewer 1. We are confused about how to respond.

MINOR COMMENTS (some of them pretty major)

1.23) When describing 3 kinds of changes after a Y-linked region stops recombining, it is odd to say “First, the X and Y show elevated genetic differentiation (measured e.g. by FST)“. The elevated differentiation is a direct result of substitutions accumulating in the Y-linked region and being restricted to the region because recombination with the homologous X-linked region does not happen (thus the Y is isolated, resulting in Y monophyly. This effect should be mentioned first, and should include Y monophyly, so there are really just 2 kinds of changes. This is not just a quibble. The authors seem unaware that detecting strata via different FST values is not independent of the Y-X divergence (though of course low diversity of Y- or X-linked sequences could also contribute to high FST, so that this measure is less reliable than divergence, and this is why the monophyly test, which relies on Y-specific variants/substitutions, often gives a clearer picture). Given that the sequences are phased, there should be no need for anything other than divergence to describe substitution accumulation in the Y-linked region, though it would be good to correct for variability among X-linked sequences, to estimate net divergence (or to mention if diversity is very low so that such a correction makes very little difference). 

As in prior rounds of reviews, Reviewer 1 again mischaracterizes our statements. The paragraph to which they are referring begins, “Three patterns of molecular variation are often seen in SDRs” (line 146). This explicitly refers to the patterns that are revealed in genomic data, not the steps of sex chromosome evolution, as Reviewer 1 implies. The three patterns are: 1) increased genetic differentiation, 2) increased male-to-female read depth ratio, and 3) X-Y monophyly. While (1) and (3) both result from lack of recombination, observations of those patterns come from very different analyses and provide independent confirmation of recombination suppression. Nowhere do we imply that *F*_ST_ and X-Y divergence as measured by *d*_S_ are independent. We note that X-Y divergence is based only on protein-coding regions, while *F*_ST_ is based on the entire genomic sequence. Consistency between the two statistics offers additional confirmation.

We object to Reviewer 1’s characterization of our knowledge of the field. For example, they imply that we are not aware that X-Y monophyly is the best method for defining SDRs, when our research group has been a leader in using X-Y monophyly for this purpose (see Dixon *et al.* 2018, Toups *et al.* 2019).

1.24) The text says “In threespine stickleback, the density of transposable elements is higher in the PAR relative to the rest of the X chromosome (Peichel, et al. 2020)”, but that paper says that there is no significant difference, so this seems misleading. If so, one cannot argue that a longer time as part of the PAR might explain why mapping rates are lowest for the younger strata on the blackspotted stickleback X.

We agree that this pattern is counterintuitive, and have expanded our hypothesis to explain it at lines 219-224. In short, the pattern of younger strata having higher *F*_ST_ is likely a bioinformatic artifact, and has been reported previously.

1.25) This low mapping rate remains a concern about accuracy.

Using whole genome sequences from four female blackspotted sticklebacks, we again observed differences in mapping rates across Chr 19 but no other chromosomes (line 208-212). This established that the different mapping rates on the X result from sex linkage and not artifacts of our cross design. Future sequencing of a blackspotted stickleback reference genome likely will shed some insight into this phenomenon, but that represents a significant undertaking which we do not believe should be required for this publication. Nearly every scientific publication raises additional questions about a system that can be addressed by more extensive research.

Regardless, the unusual pattern only affects read depth ratio, which is one of several analyses supporting the existence of different strata. Downstream filtering by mean read depth removes loci affected by X mapping issues from our analyses that rely on *d*_S_ or gene trees.

1.26) It is suggested that sexually antagonistic selection can favor the maintenance of ancestral sex chromosomes (impeding turnovers), bwithout any explanation. The concept should be explained briefly.

We added a brief explanation at lines 372-374.

1.27) I believe that the spinach example mentioned at the end of the ms involved a sex chromosome-autosome fusion, and therefore that rates of degeneration many not be comparable. The authors should check this carefully. I also believe that Y-X divergence results give no clear conclusion about the ages of the Rumex hastatulus and R. rothschildianus Ys, so the statement that they “are of similar age” is not justified. The reported extreme difference in sex chromosome degeneration between two closely related poeciliid fish lineages reported by Darolti, et al. (2019) is again explained in terms of times of recombination suppression, not a difference in degeneration rates like the stratum S1 difference in the sticklebacks, and this should be made clear.

Again, Reviewer 1 is mistaken. The spinach study (Fujito *et al.* 2015) showed that in two closely-related species, one has heteromorphic sex chromosomes and the other has homomorphic sex chromosomes. This is analogous to the guppy system (see below), except that they were also able to conclusively demonstrate that the Y chromosomes of the two are homologous via the use of Y-specific genetic markers. No fusion is mentioned in that paper, or other recent papers on the spinach sex chromosomes. Even if it did, a fusion is also present in blackspotted sticklebacks, making it directly comparable.

The study of *Rumex* (Crowson *et al.* 2017), explicitly quantified the age of the SDR in *R. hastatulus* (8–11 million generations ago) and *R. rothschilidianus* (9–16 million generations ago). We have clarified that the SDRs of the Ys are of similar age (459-461).

We added a sentence to the Discussion noting that the studies we cited did not compare rates of Y degeneration between homologous strata. This distinction emphasizes the novelty of our study, which also identified differences between homologous strata.

1.28) The guppy XY pair are not completely non-recombining see Bergero et al. 2019 (doi: 10.1073/pnas.1818486116), but the homologous pair in *Poecilia* (or Micropoecilia) have done so. It is odd to write that the authors cannot confirm that the highly degenerate Y chromosome of Poecilia picta is homologous to the undegenerate (non-degenerate) Ys of its congeners (i.e. the guppy), as all, or almost all guppy LG12 genes that have been tested are detectable on the X of P. picta, but are hemizygous in males (except for a few PAR genes), so the chromosome as a whole is certainly homologous. This is in an unpublished paper (doi: 10.2139/ssrn.3417937), but is consistent with the genome sequence results of Darolti et al..

As noted in our last response to reviewers, this reviewer is using a different definition of “homologous sex chromosomes” than ours. The term is ambiguous and does not currently have a standard definition. We therefore included an explicit definition at lines 100-104 that we apply throughout our paper, and which we believe better satisfies the conventional definition of “homology”. This reviewer is correct that there is no question that the Y chromosomes of *P. picta* and *P. reticulata* evolved from the same autosome. When we say that we cannot confirm that they are homologous, we mean that we cannot confirm that they both descend from the same chromosome that carried the original male-determining mutation. There is no evidence in the literature refuting the alternative hypothesis that the Ys evolved independently from the same autosome. In contrast, we were explicitly able to disprove that hypothesis in sticklebacks.

**Reviewer 2**

2.1) I was pleased to see this interesting paper again. It was already a solid study and has been improved with more quantitative and better-documented methods, as well as a framing that fits the data better. This study uses whole-genome sequencing to identify the sex chromosome and SDR of the blackspine stickleback (Gasterosteus wheatlandi), with the goal of more fully comparing rates of sex chromosome divergence and degeneration across all three species in the genus. It first establishes that chromosome 19 is an ancient sex chromosome in G. wheatlandi, with a high proportion of hemizygous and highly diverged sequence, elevated Fst between males and females, an elevated fraction of monophyletic gene trees, and four distinct strata of recombination suppression. Chromosome 12 is then identified as a fused neo-sex chromosome, with a recent origin and two possible strata. Finally, the authors argue for a shared origin of chromosome 19 as the sex chromosome in the common ancestor of all three Gasterosteus species, based on two approaches: a clever novel method for identifying shared Y-specific duplications from autosomes, and comparisons of gene trees in different regions of the genome. The shared duplication approach is likely to have many applications in other systems. Finally, the discussion unpacks well some hypotheses that might account for the dramatically different rates of divergence in these species, tying the observations to theory. 
 In general, the article is compelling and well-argued. I would suggest a couple of changes to the presentation to make the story clearer, but they are largely cosmetic.

We thank Reviewer 2 for their enthusiasm and for their useful suggestions.

2.2) L 43-46 I think this could still be sharpened a bit. The argument pivots from dramatic differences across taxa (extreme degeneration over 140 million years vs. minimal degeneration over 1 million years) to comparisons within taxa to possible explanations. Having a range of examples that covers more of the variation (than those two poles - e.g., there are some old homomorphic and minimally diverged systems, and some rapidly diverging ones, particularly in plants) might make more pointed the question of "why so much variation." Phylogenetic effects might be less relevant for comparisons of closely related species, though of course could be in play. And there are probably more options than "age, phylogenetic effects, or it's unpredictable" - what about selection? Maybe rewrite towards to "demonstrating this kind of dramatic variation even within closely related groups is necessary for identifying the selective forces behind variable evolutionary rates in sex chromosomes" or something like that.

We have expanded the introduction as suggested to provide more examples of prior studies demonstrating variation in the outcomes of sex chromosome evolution, and we have clarified the specific questions that we addressed in our study (lines 91-99).

2.3) 54 in all three species?

That is correct. We noted that these three species represent all currently recognized species in the genus (line 66).

2.4) 78ff If I understand that correctly, chr 12 being the ancestral chromosome is a less parsimonious explanation, given that the tip states are (12+19,(19,19+9)).

We agree that parsimony within *Gasterosteus* suggests that Chr 19 is ancestral, but Chr 12 is the ancestral sex chromosome in the outgroup genus *Pungitius* (line 96) and rates of sex chromosome turnover are high in sticklebacks (lines 56-62). Our results provide unambiguous evidence that Chr 19 is indeed the ancestral sex chromosome of *Gasterosteus*, which we believe is preferable to inferences based on parsimony.

2.5) 142ff and figure 2: I'm confused about Fst in figure 2b. In the text, and the figure, Fst is low in the pseudoautosomal region (so far, so good), but it actually appears to be higher in the less diverged strata with less skewed read depth ratios (S3 and S4) than in S1 and S2. I'm guessing this is due to Fst's sensitivity to absolute diversity (which the gene monophyly analysis establishes is low) and recombination rate (obviously suppressed here), but perhaps comment?

This is now explained at lines 219-224. When a stratum forms, *F*_ST_ initially increases as the X and Y began to diverge. Eventually the Y diverges from the X so much that Y-linked reads fail to map to the X reference, resulting in hemizygous sites that SNP calling programs assume are homozygous, causing *F*_ST_ to decrease.

2.6) Passim: comma use in citations inconsistent (name et al. vs name, et al)

Fixed.

2.7) A supplemental figure of the crossing design and phasing would be helpful, just to describe the pipeline visually. It took me a few reads to grasp that the haplotypes were being reconstructed independently, and the interspecific crosses facilitated phasing of those independent haplotypes. A figure would have made it clearer faster.

Thanks for the suggestion. We added Supplementary Fig. 1 showing the experimental design.

2.8) A major point of the paper is that the extent of differentiation differs considerably among the three species' sex chromosomes; this is partly shown in Figure 1 (not mentioned in the text). Maybe rearrange Figure 1 to also show the Japanese Stickleback's karyotype? And perhaps a few other aspects of their structures, like possible inversions or degrees of differentiation between X and Y in the strata of different species or age estimates? Maybe Fig. 1 and Fig. 7 could be effectively combined into a remade Fig. 7?

Excellent suggestion, thank you! Please see the new Figure 1.

2.9) Figure 1 is not currently referenced in the text.

Fig. 1 is first cited on line 63, and many times throughout the paper.

2.10) Figures 5 and 6 could be combined - I think they'd be clearer as one figure - and perhaps either the colored inset boxes or the strata could also have numbers of topologies attached? It would be nice to know the numerators and denominators of "genes that match this topology / total genes."

We tried combining Figs. 5 and 6 (now Figs. 2 and 6) and found the result was too confusing to understand. We added Supplemental Table 1 with the number of trees that match each topology, broken down by strata.
