# Supplementary material for: Heterogeneous Histories of Recombination Suppression on Stickleback Sex Chromosomes
Source: Mol Biol Evol. 2021 Jun 12;38(10):4403–18. doi: 10.1093/molbev/msab179 (PMC8476171; doi:10.1093/molbev/msab179)
Supplement: msab179_Supplementary_Data [file msab179_supplementary_data.zip › Sardell MBE supp mat.pdf]

Supplementary table

Supplementary table 1: Number of trees with the topologies shown in fig. 6 and supplementary fig. 7, broken down by strata. BS = blackspotted, JS = Japan Sea, TS = threespine

	Chr 19					Chr 12		
Region	PAR	R3	R2	R1	S1	R4	R5	PAR
Homologous Ys	0	0	0	0	7	0	0	0
Turnover: JS/TS X>Y	0	0	0	0	2	0	0	0
Turnover: BS X>Y	0	0	0	6	0	0	0	0
Independent origins	0	0	11	29	3	0	0	0
Old SDR in BS, PAR in JS	0	12	1	0	0	13	18	0
New SDR in BS, PAR in JS	0	0	0	0	0	7	123	0
PAR in both	1	3	0	0	0	1	0	42
Other topologies	0	0	0	9	9	0	0	0

Supplementary figure captions

Supplementary fig. S1. The experimental design used to phase sequences from X and Y chromosomes. A blackspotted male (blue X chromosomes and orange Y chromosomes) is crossed to a threespine female (green X chromosomes). Whole genome sequences are obtained from both parents, one hybrid son, and one hybrid daughter. If a read in the daughter maps to the sex chromosome of the threespine reference genome and matches a read in the father but not the mother, it is from the blackspotted X chromosome inherited from her father. Likewise, a read from the sex chromosome of the son that matches a read in the father but not the mother is from the blackspotted Y chromosome. Using this logic, we obtain phased sequences of the X and Y chromosomes in the blackspotted father. We carried out this strategy on 15 families to obtain sequences of 15 X chromosomes and 15 Y chromosomes from the blackspotted stickleback.

Supplementary fig. S2. Read depth statistics for 15 sons and 15 daughters from the pedigrees. Histogram of male/female read depth ratio for all SNPs on the two pairs of sex chromosomes and the autosomes. Dashed vertical lines show values expected with Y chromosomes that are highly degenerated (ratio = 0.5) and non-degenerated (ratio = 1).

Supplementary fig. S3: Mean read depth in sons and daughters for Chr 19 (top) and Chr 12 (bottom). Dots show averages in 10 Kb windows. Dashed vertical lines represent boundaries between the PARs (labeled P) and the strata in the SDR (labeled S1 and R1 to R5) that were identified using methods described in text. Solid horizontal lines show the mean for each stratum. The circles on the X axis show the locations of the centromeres on Chr 19 and Chr 12 in threespine stickleback.

Supplementary fig. S4: Divergence statistics for individual genes on Chr 19 (left panels) and Chr 12 (right panels). Top panels: divergence at nonsynonymous sites (d_N) between blackspotted stickleback (BS) X and Y chromosomes. Bottom panels: d_N/d_S between blackspotted stickleback (BS) X and Y chromosomes. Only genes containing at least 5 SNPs (Chr 19) or 8 SNPs (Chr 12) are included to avoid division by zero errors. Dashed vertical lines represent boundaries between the PARs (labeled P) and the strata in the SDR (S1 and R1 to R5) that were identified using methods described in text. Solid horizontal lines show the mean for each stratum. The circles on the X axis of the lower panel show the locations of the centromeres on Chr 19 and Chr 12 in threespine stickleback.

Supplementary fig. S5: Violin plots showing the distributions of d_N and d_S values in the strata on the ancestral sex chromosomes (Chr 19) and the neo-sex chromosomes (Chr 12) based on comparisons

between the X and Y in blackspotted stickleback. The dots show the means and the vertical lines show +/– a standard deviation. Note that the scales of the vertical axes differ between the panels.

Supplementary fig. S6: Read depth statistics provide evidence for autosome-to-Y duplications in the three regions of the blackspotted SDR shown in Fig. 5. Each point represents a SNP with $F_{ST} > 0.25$ and a male-specific allele in both blackspotted and Japan Sea sticklebacks. Points denote SNPs from the 10 Kb windows with the shapes shown in the key. Left panel: Male/female read depth ratio in blackspotted (BS) sticklebacks (left, purple) and Japan Sea (JS) sticklebacks (right, gray). Solid horizontal gray line (near bottom) indicates the autosomal mean. Horizontal dashed gray lines indicate intervals of 0.5 times the autosomal mean, *i.e.* the expected values for one or more autosome-to-Y duplications. Right panel: Fraction of reads with a male-specific allele in blackspotted (BS) sticklebacks (left, purple) and Japan Sea (JS) sticklebacks (right, gray). Solid horizontal gray line (near top) indicates the value expected for non-duplicated regions.

Supplementary fig. S7: Multispecies gene tree topologies along Chr 12 demonstrate the recent origin of the SDR on the neo-sex chromosome. Each dot represents the maximum-likelihood topology for a 100 Kb window. Representative trees are shown at right (BS = blackspotted, JS = Japan Sea, TS = threespine). In most windows from the SDR, a monophyletic clade of blackspotted stickleback neo-Ys is imbedded within the blackspotted neo-Xs. The circle on the X axis of the lower panel shows the location of the centromere in threespine stickleback. Supplementary table 1 shows how many genes trees were of each topology in the two strata.

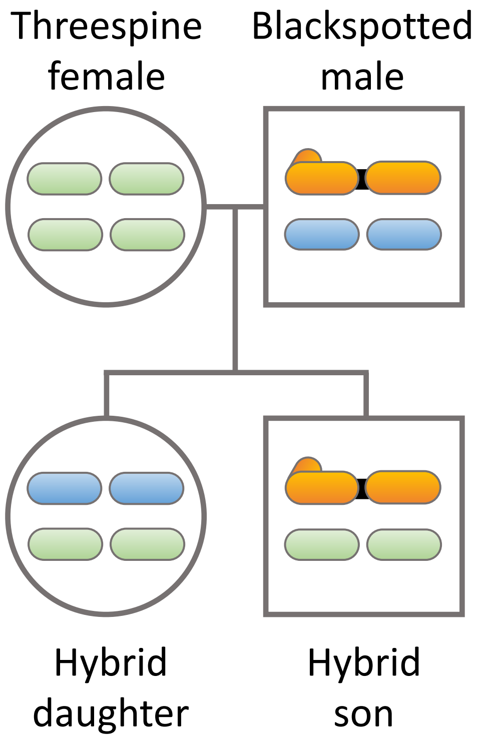
Threespine
female
Blackspotted
male
Hybrid
daughter
Hybrid
son

Supp. Fig. S1

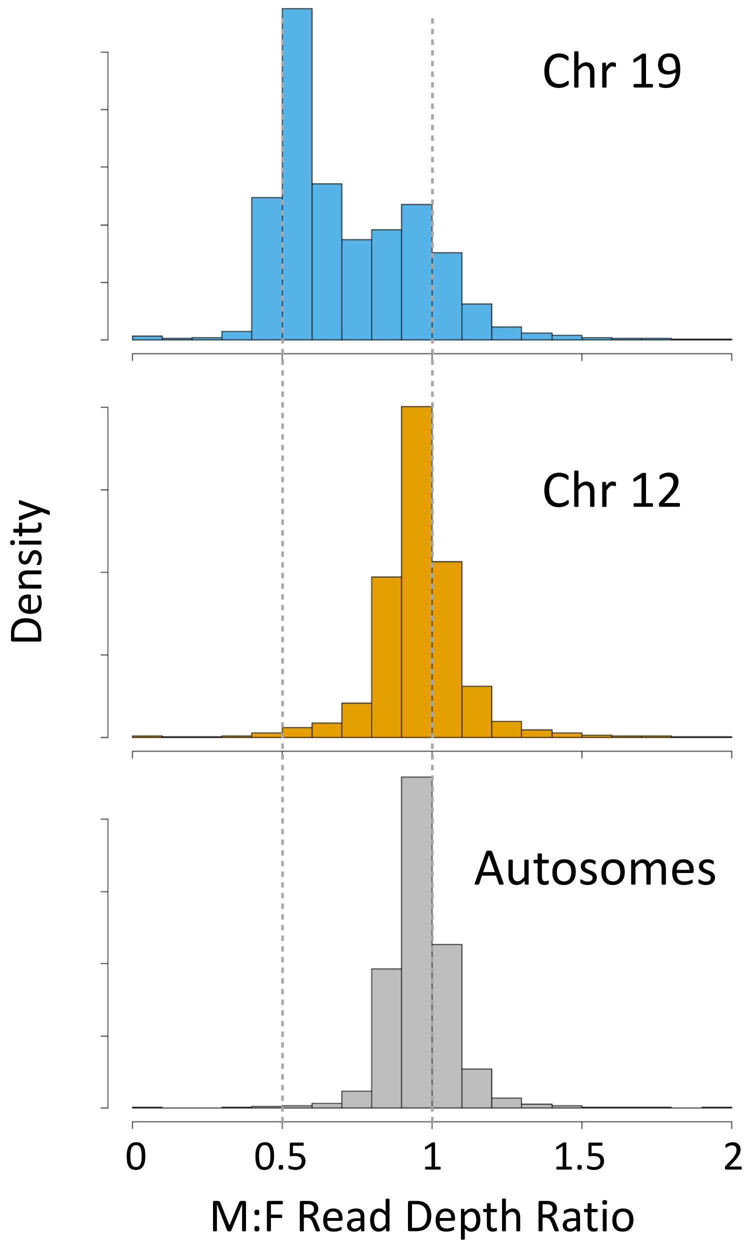

Supp. Fig. S2

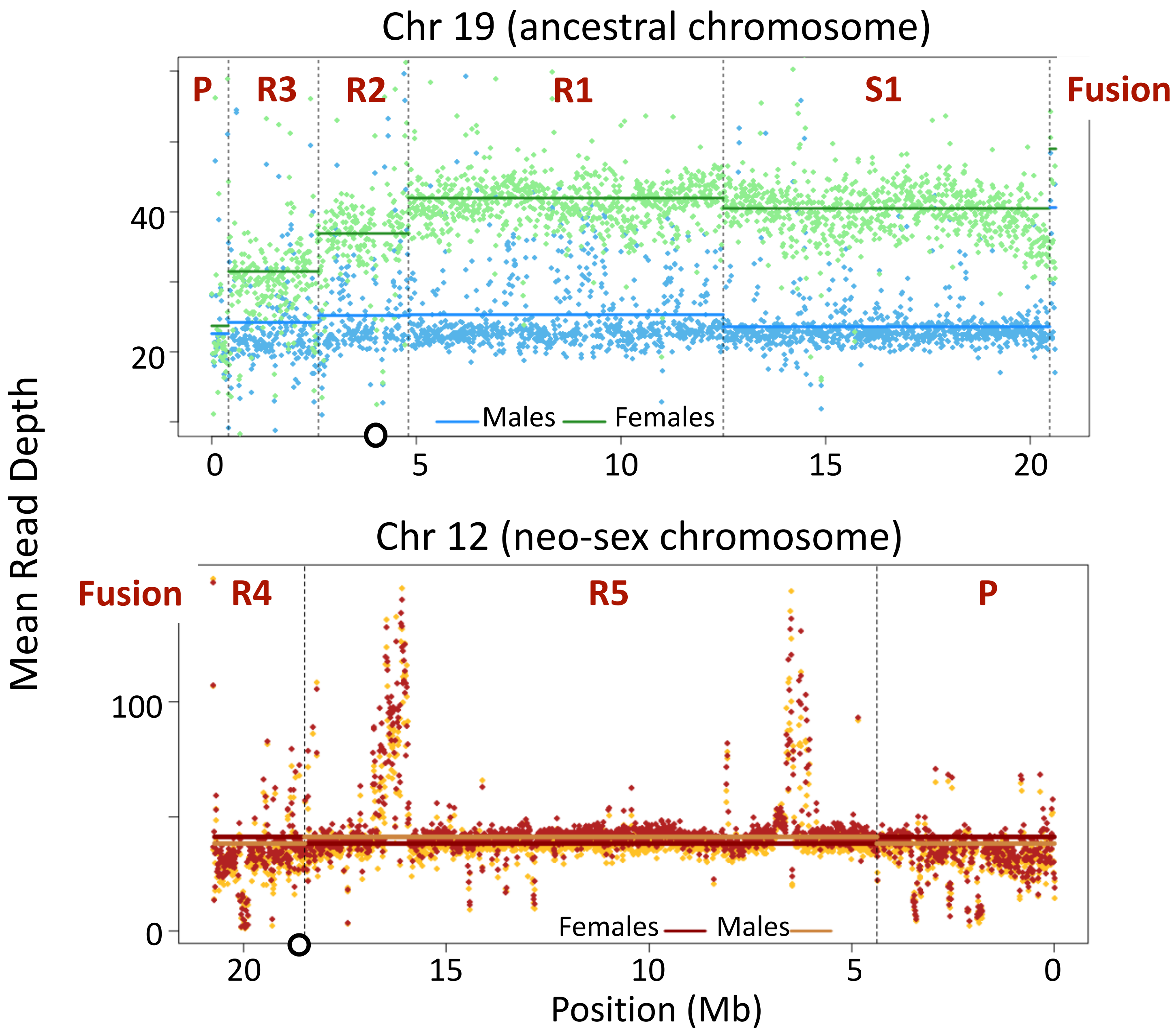
Chr 19 (ancestral chromosome)
P
R3
R2
R1
S1
Fusion
40
20
Males
Females
0
5
10
15
20
Mean Read Depth
Chr 12 (neo-sex chromosome)
Fusion
R4
R5
P
100
0
Females
Males
20
15
10
5
0
Position (Mb)

Supp. Fig. S3

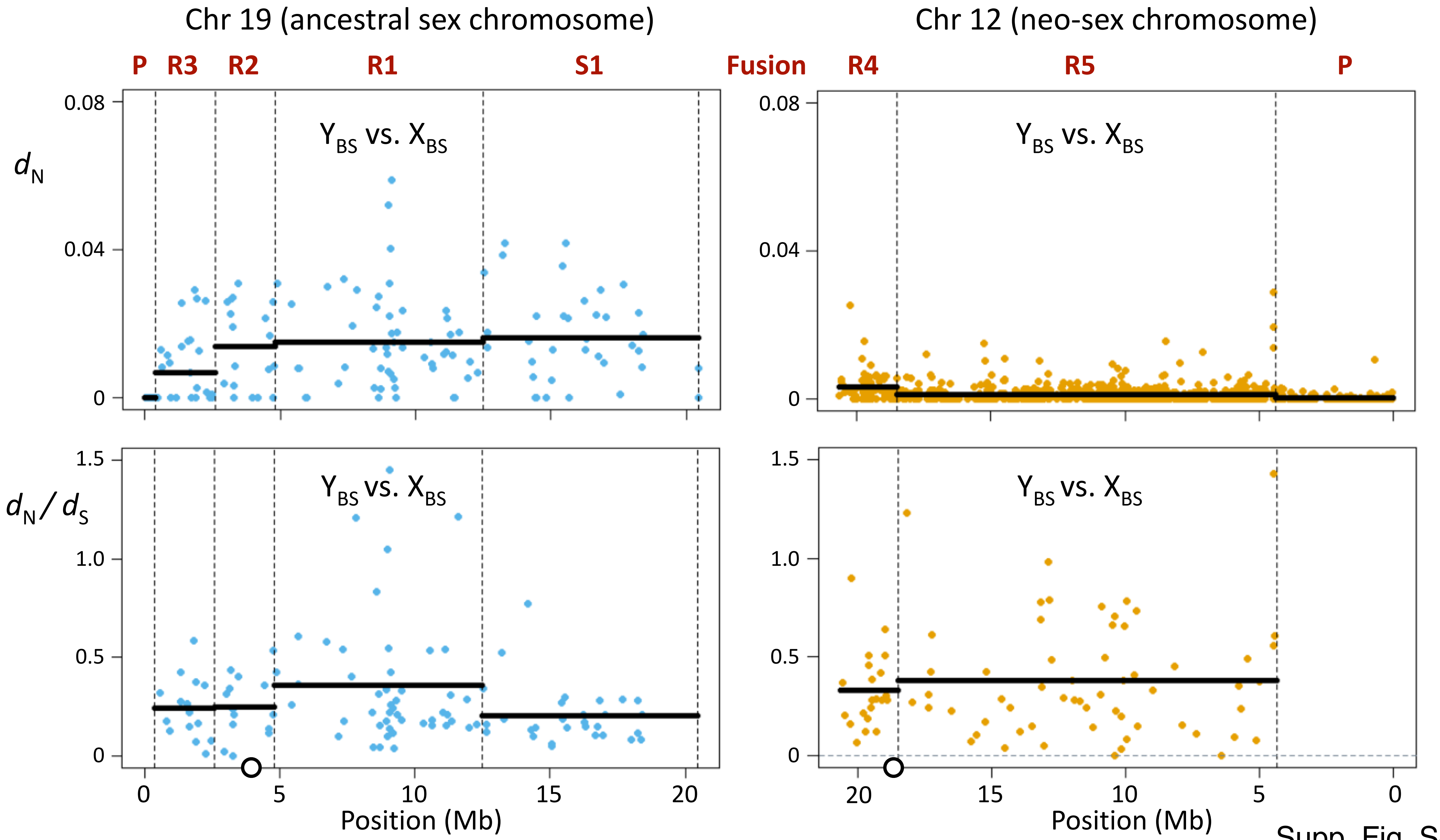
Chr 19 (ancestral sex chromosome)
Chr 12 (neo-sex chromosome)
P
R3
R2
R1
S1
Fusion
R4
R5
P
d_N
d_N / d_S
Y_{BS} vs. X_{BS}
Y_{BS} vs. X_{BS}
Y_{BS} vs. X_{BS}
Y_{BS} vs. X_{BS}
0.08
0.04
0
1.5
1.0
0.5
0
0 5 10 15 20
20 15 10 5 0
Position (Mb)
Position (Mb)

Supp. Fig. S4

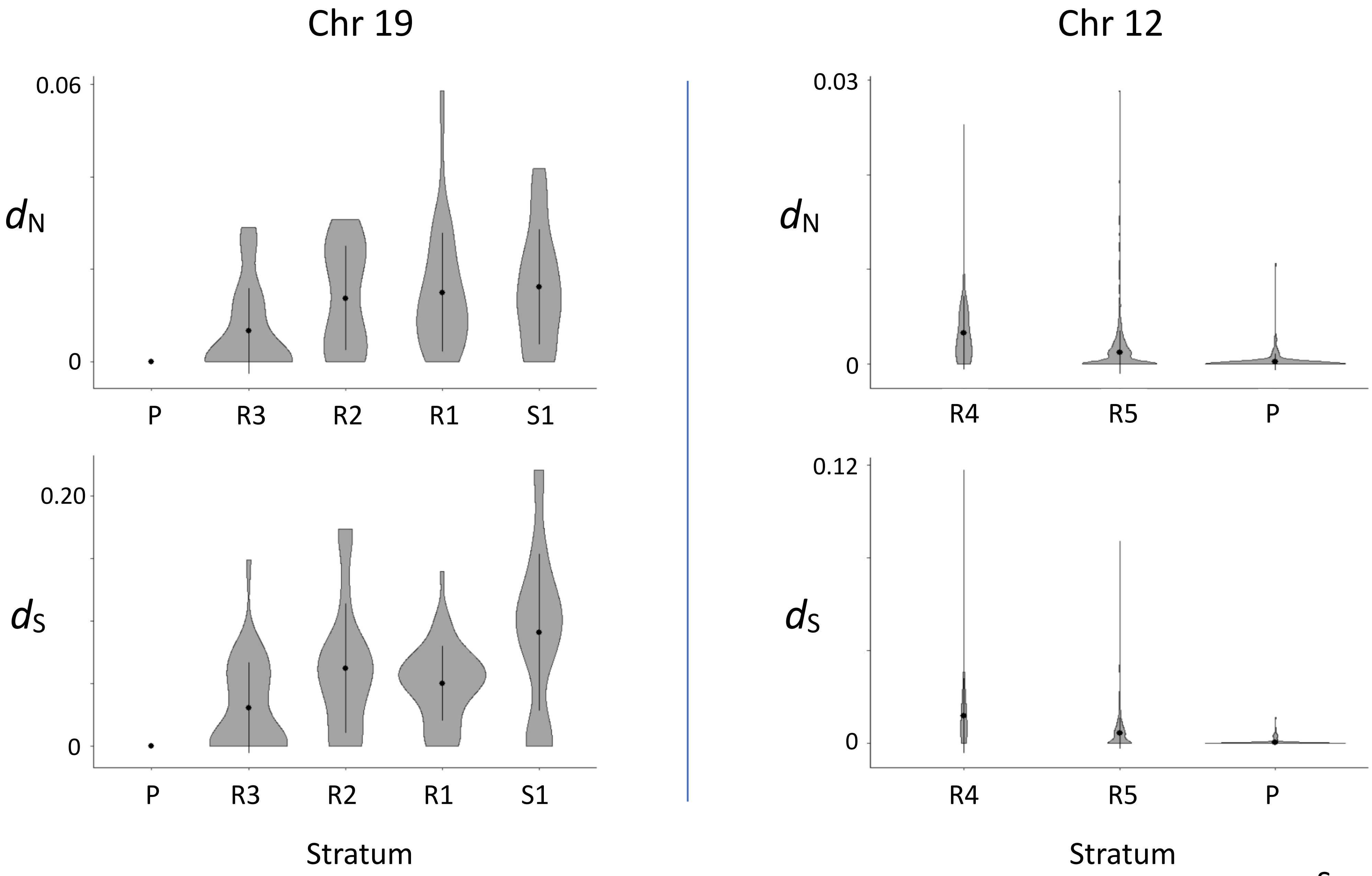

Supplementary fig. S5

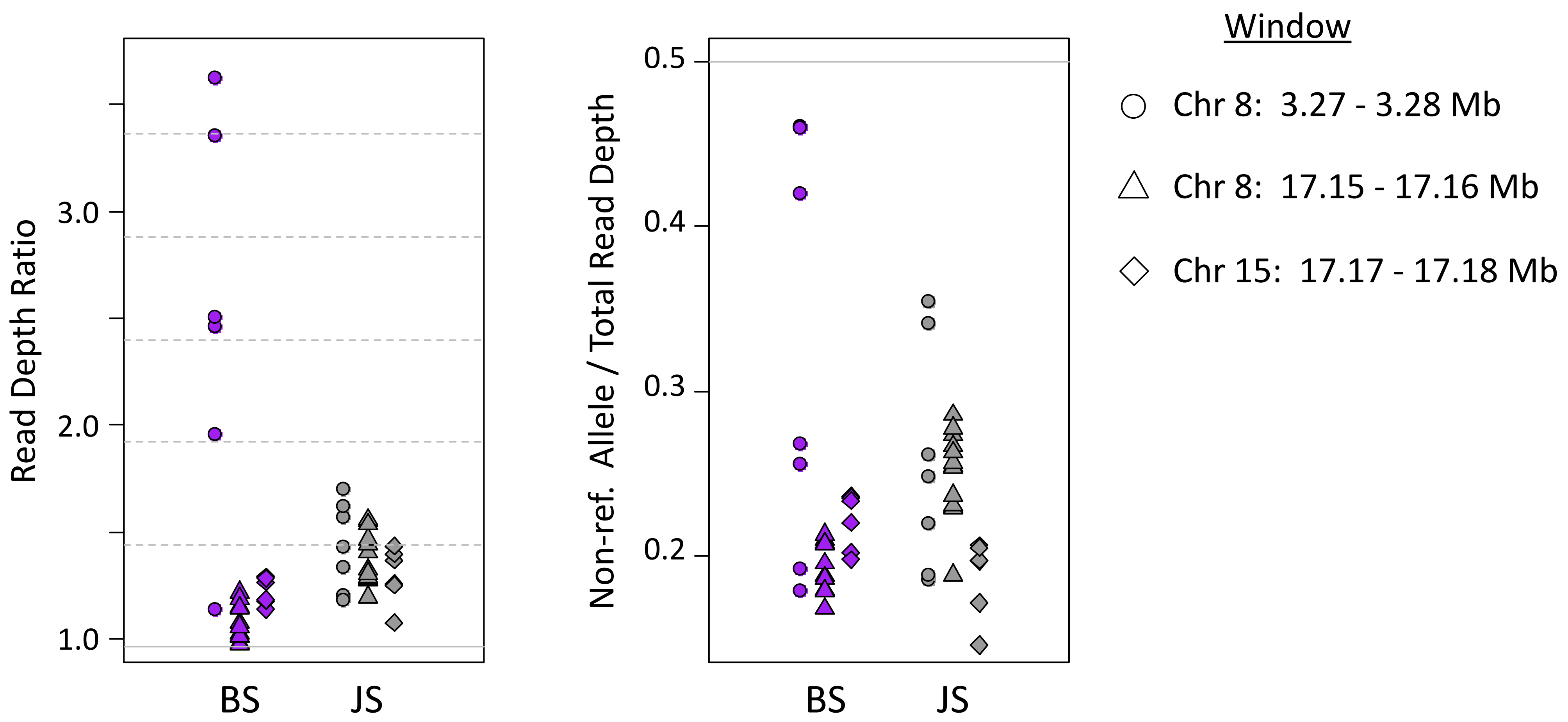

Read Depth Ratio
1.0
2.0
3.0
BS
JS
Non-ref. Allele / Total Read Depth
0.2
0.3
0.4
0.5
Window
Chr 8: 3.27 - 3.28 Mb
Chr 8: 17.15 - 17.16 Mb
Chr 15: 17.17 - 17.18 Mb

Supp. Fig. S6

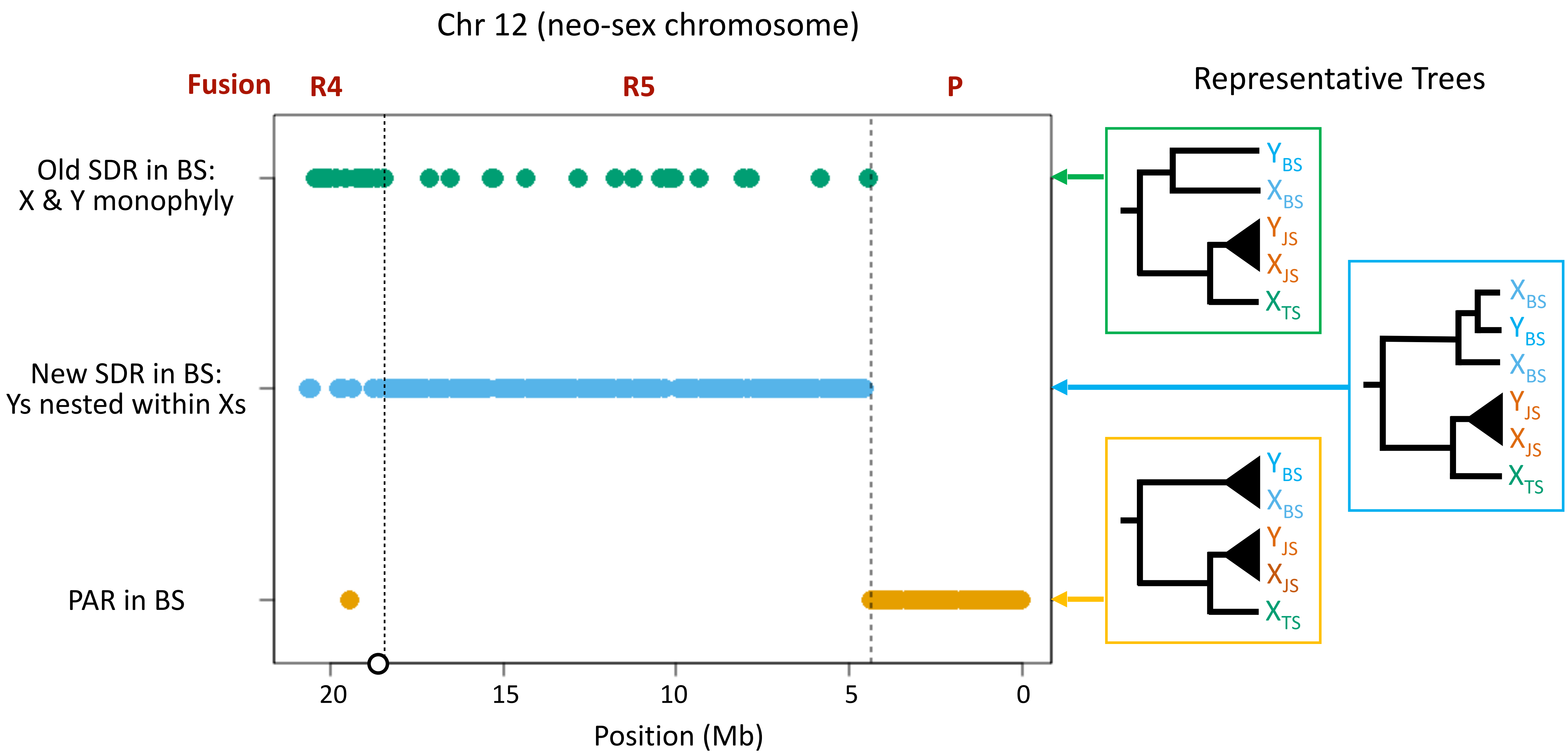

Chr 12 (neo-sex chromosome)
Fusion
R4
R5
P
Representative Trees
Old SDR in BS:
X & Y monophyly
New SDR in BS:
Ys nested within Xs
PAR in BS
20
15
10
5
0
Position (Mb)
YBS
XBS
YJS
XJS
XTS
XBS
YBS
XBS
YJS
XJS
XTS
YBS
XBS
YJS
XJS
XTS

Supp. Fig. S7
